# Supplementary material for: Number and timing of primary cleft lip and palate repair surgeries in England: whole nation study of electronic health records before and during the COVID-19 pandemic
Source: BMJ Open. 2023 Jun 13;13(6):e071973. doi: 10.1136/bmjopen-2023-071973 (PMC10276964; doi:10.1136/bmjopen-2023-071973)
Supplement: Supplementary data [file bmjopen-2023-071973supp001.pdf]

SUPPLEMENTARY MATERIALS

Supplementary Figure 1: Flow chart showing inclusion criteria into the study

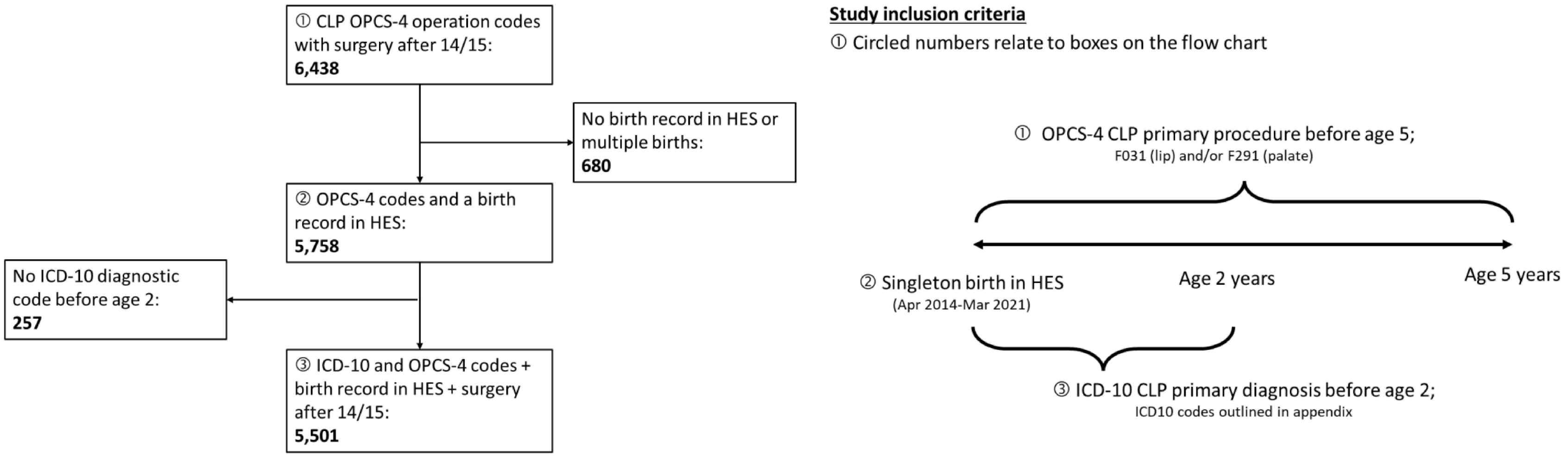

Supplementary information 1: OPCS-4 CLP surgery procedure code lists

OPCS-4 code	Surgery type
F031	Primary closure of cleft lip
F291	Primary palate repair

Supplementary information 2: Cleft lip and palate ICD-10 codes

ICD-10 codes	Cleft type
Q35x	Cleft lip
Q36x	Cleft palate
Q371, Q373, Q375, Q379	Unilateral cleft lip and palate
Q370, Q372, Q374, Q378	Bilateral cleft lip and palate

Etoori D, *et al. BMJ Open* 2023; 13:e071973. doi: 10.1136/bmjopen-2023-071973

Supplementary information 3: Congenital anomalies code list

Code	Description
	Congenital malformations of the nervous system
Q00	Anencephaly and similar malformations
Q01	Encephalocele
Q02	Microcephaly
Q03	Congenital hydrocephalus
Q04	Other congenital malformations of brain
Q05	Spina bifida
Q06	Other congenital malformations of spinal cord
Q07	Other congenital malformations of nervous system
	Congenital malformations of eye, ear, face and neck
Q10	Congenital ptosis
Q11	Anophthalmos, microphthalmos and macrophthalmos
Q12	Congenital lens malformations
Q13	Congenital malformations of anterior segment of eye
Q14	Congenital malformations of posterior segment of eye
Q15	Other congenital malformations of eye
Q16	Congenital malformations of ear causing impairment of hearing
Q17	Other congenital malformations of ear

Etoori D, *et al. BMJ Open* 2023; 13:e071973. doi: 10.1136/bmjopen-2023-071973

Q18	Other congenital malformations of face and neck
	Congenital malformations of the circulatory system
Q20	Congenital malformations of cardiac chambers and connections
Q21	Congenital malformations of cardiac septa
Q22	Congenital malformations of pulmonary and tricuspid valves
Q23	Congenital malformations of aortic and mitral valves
Q24	Other congenital malformations of heart
Q25	Congenital malformations of great arteries
Q26	Congenital malformations of great veins
Q27	Other congenital malformations of peripheral vascular system
Q28	Other congenital malformations of circulatory system
	Congenital malformations of the respiratory system
Q30	Congenital malformations of nose
Q31	Congenital malformations of larynx
Q32	Congenital malformations of trachea and bronchus
Q33	Congenital malformations of lung
Q34	Other congenital malformations of respiratory system
	Other congenital malformations of the digestive system
Q38	Other congenital malformations of tongue, mouth and pharynx
Q39	Congenital malformations of oesophagus
Q40	Other congenital malformations of upper alimentary tract
Q41	Congenital absence, atresia and stenosis of small intestine

Etoori D, *et al. BMJ Open* 2023; 13:e071973. doi: 10.1136/bmjopen-2023-071973

Q42	Congenital absence, atresia and stenosis of large intestine
Q43	Other congenital malformations of intestine
Q44	Congenital malformations of gallbladder, bile ducts and liver
Q45	Other congenital malformations of digestive system
	Congenital malformations of the genital organs
Q50	Congenital malformations of ovaries, fallopian tubes and broad ligaments
Q51	Congenital malformations of uterus and cervix
Q52	Other congenital malformations of female genitalia
Q53	Undescended testicle
Q54	Hypospadias
Q55	Other congenital malformations of male genital organs
Q56	Indeterminate sex and pseudohermaphroditism
	Congenital malformations of the urinary system
Q60	Renal agenesis and other reduction defects of kidney
Q61	Cystic kidney disease
Q62	Congenital obstructive defects of renal pelvis and congenital malformations of ureter
Q63	Other congenital malformations of kidney
Q64	Other congenital malformations of urinary system
	Congenital malformations and deformations of the musculoskeletal system
Q65	Congenital deformities of hip
Q66	Congenital deformities of feet
Q67	Congenital musculoskeletal deformities of head, face, spine and chest

Q68	Other congenital musculoskeletal deformities
Q69	Polydactyly
Q70	Syndactyly
Q71	Reduction defects of upper limb
Q72	Reduction defects of lower limb
Q73	Reduction defects of unspecified limb
Q74	Other congenital malformations of limb(s)
Q75	Other congenital malformations of skull and face bones
Q76	Congenital malformations of spine and bony thorax
Q77	Osteochondrodysplasia with defects of growth of tubular bones and spine
Q78	Other osteochondrodysplasias
Q79	Congenital malformations of the musculoskeletal system, not elsewhere classified
	Other congenital malformations
Q80	Congenital ichthyosis
Q81	Epidermolysis bullosa
Q82	Other congenital malformations of skin
Q83	Congenital malformations of breast
Q84	Other congenital malformations of integument
Q85	Phakomatoses, not elsewhere classified
Q86	Congenital malformation syndromes due to known exogenous causes, not elsewhere classified
Q87	Other specified congenital malformation syndromes affecting multiple systems
Q89	Other congenital malformations, not elsewhere classified

Etoori D, *et al. BMJ Open* 2023; 13:e071973. doi: 10.1136/bmjopen-2023-071973

	Chromosomal abnormalities, not elsewhere classified
Q90	Down syndrome
Q91	Edwards syndrome and Patau syndrome
Q92	Other trisomies and partial trisomies of the autosomes, not elsewhere classified
Q93	Monosomies and deletions from the autosomes, not elsewhere classified
Q95	Balanced rearrangements and structural markers, not elsewhere classified
Q96	Turner syndrome
Q97	Other sex chromosome abnormalities, female phenotype, not elsewhere classified
Q98	Other sex chromosome abnormalities, male phenotype, not elsewhere classified
Q99	Other chromosome abnormalities, not elsewhere classified

Etoori D, *et al. BMJ Open* 2023; 13:e071973. doi: 10.1136/bmjopen-2023-071973
