# Supplementary material for: Number and timing of primary cleft lip and palate repair surgeries in England: whole nation study of electronic health records before and during the COVID-19 pandemic
Source: BMJ Open. 2023 Jun 13;13(6):e071973. doi: 10.1136/bmjopen-2023-071973 (PMC10276964; doi:10.1136/bmjopen-2023-071973)
Supplement: Author's manuscript [file bmjopen-2023-071973.draft_revisions.pdf]

BMJ Open is committed to open peer review. As part of this commitment we make the peer review history of every article we publish publicly available.

When an article is published we post the peer reviewers' comments and the authors' responses online. We also post the versions of the paper that were used during peer review. These are the versions that the peer review comments apply to.

The versions of the paper that follow are the versions that were submitted during the peer review process. They are not the versions of record or the final published versions. They should not be cited or distributed as the published version of this manuscript.

BMJ Open is an open access journal and the full, final, typeset and author-corrected version of record of the manuscript is available on our site with no access controls, subscription charges or pay-per-view fees (http://bmjopen.bmj.com).

If you have any questions on BMJ Open's open peer review process please email info.bmjopen@bmj.com

BMJ Open

Number and timing of primary cleft lip and palate repair surgeries in England: whole nation study of electronic health records before and during the COVID-19 pandemic.

Journal:	*BMJ Open*
Manuscript ID	bmjopen-2023-071973
Article Type:	Original research
Date Submitted by the Author:	19-Jan-2023

Complete List of Authors:	Etoori, David; University College London Institute of Health Informatics<br>Park, Min Hae; London School of Hygiene & Tropical Medicine<br>Blackburn, Ruth; University College London Institute of Health Informatics<br>Fitzsimons, Kate; Royal College of Surgeons of England<br>Butterworth, Sophie; Royal College of Surgeons of England<br>Medina, Jibby; Royal College of Surgeons of England, Clinical Effectiveness Unit<br>Mc Grath-Lone, Louise; University College London Institute of Education<br>Russell, Craig; Queen Elizabeth University Hospital<br>van der Meulen, Jan; London School of Hygiene & Tropical Medicine

Keywords:	COVID-19, Paediatric surgery < SURGERY, EPIDEMIOLOGY

SCHOLARONE™
Manuscripts

I, the Submitting Author has the right to grant and does grant on behalf of all authors of the Work (as defined in the below author licence), an exclusive licence and/or a non-exclusive licence for contributions from authors who are: i) UK Crown employees; ii) where BMJ has agreed a CC-BY licence shall apply, and/or iii) in accordance with the terms applicable for US Federal Government officers or employees acting as part of their official duties; on a worldwide, perpetual, irrevocable, royalty-free basis to BMJ Publishing Group Ltd ("BMJ") its licensees and where the relevant Journal is co-owned by BMJ to the co-owners of the Journal, to publish the Work in this journal and any other BMJ products and to exploit all rights, as set out in our licence.

The Submitting Author accepts and understands that any supply made under these terms is made by BMJ to the Submitting Author unless you are acting as an employee on behalf of your employer or a postgraduate student of an affiliated institution which is paying any applicable article publishing charge ("APC") for Open Access articles. Where the Submitting Author wishes to make the Work available on an Open Access basis (and intends to pay the relevant APC), the terms of reuse of such Open Access shall be governed by a Creative Commons licence – details of these licences and which Creative Commons licence will apply to this Work are set out in our licence referred to above.

Other than as permitted in any relevant BMJ Author's Self Archiving Policies, I confirm this Work has not been accepted for publication elsewhere, is not being considered for publication elsewhere and does not duplicate material already published. I confirm all authors consent to publication of this Work and authorise the granting of this licence.

For peer review only

Title: Number and timing of primary cleft lip and palate repair surgeries in England: whole nation study of electronic health records before and during the COVID-19 pandemic.

Authors: David Etoori[1], Min Hae Park[2], Ruth Blackburn[1*], Kate Fitzsimons,[3] Sophie Butterworth,[3] Jibby Medina[3], Louise Mc Grath-Lone[1], Craig Russell[4], Jan Van Der Meulen[2]

Affiliations:

1. University College London Institute of Health Informatics, 222 Euston Road, London, NW1 2DA, UK.
2. London School of Hygiene and Tropical Medicine, Keppel Street, London, WC1E 7HT, UK.
3. Clinical Effectiveness Unit, Royal College of Surgeons of England, 38 – 43 Lincoln's Inn Fields, London, UK.
4. Royal Hospital for Children, Queen Elizabeth University Hospital, Glasgow, UK

*** Corresponding author:**

Dr Ruth Blackburn, 222 Euston Road, London, NW1 2DA, UK, Email: r.blackburn@ucl.ac.uk

Email addresses: DE: d.etoori@ucl.ac.uk**;** MHP: MinHae.Park@lshtm.ac.uk; RB: r.blackburn@ucl.ac.uk; KF: kfitzsimons@rcseng.ac.uk**;** SB: sbutterworth@rcseng.ac.uk**;** JM; jmedina@rcseng.ac.uk; LMcGL: l.mcgrath-lone@ucl.ac.uk**;** CR: craig.russell@ggc.scot.nhs.uk**;** JVDM: Jan.vanderMeulen@lshtm.ac.uk

Key words: COVID-19; orofacial cleft, cleft lip; cleft palate; timing of surgery

For peer review only - http://bmjopen.bmj.com/site/about/guidelines.xhtml

Word count: Abstract 226; Text 2284

ABSTRACT

Objective To quantify differences in number and timing of first primary cleft lip and palate (CLP) repair procedures during the first year of the COVID-19 pandemic (1st April 2020 to 31st March 2021; 2020/21) compared to the preceding year (1st April 2019 to 31st March 2020; 2019/21).

Design National study of administrative hospital data.

Setting National Health Service hospitals in England.

Study population Children <5 years undergoing primary repair for an orofacial cleft (OPCS-4 codes F031, F291)

Main exposure Procedure date (2020/21 vs 2019/20)

Main outcomes Numbers and timing (age in months) of first primary CLP procedures.

Results 1,716 CLP primary repair procedures were included in the analysis. In 2020/21, 774 CLP procedures were carried out compared to 942 in 2019/20, a reduction of 17.8 % (95% confidence interval 9.5% to 25.4%). The reduction varied over time in 2020/21, with no surgeries at all during the first two months (April and May 2020). Compared to 2019/20, first primary lip repair procedures performed in 2020/21 were delayed by 1.6 months on average (95% confidence interval 0.9 to 2.2 months). Delays in primary palate repairs were smaller on average but varied across the nine geographical regions.

Conclusion There were significant reductions in the number and delays in timing of first primary CLP repair procedures in England during the first year of the pandemic, which may affect long-term outcomes.

For peer review only - http://bmjopen.bmj.com/site/about/guidelines.xhtml

INTRODUCTION

Around 1 in 670 children in the England, Wales and Northern Ireland are born alive with an orofacial cleft that may affect only the lip, only the palate, or both. (1) An orofacial cleft can have significant effects on children's lives, including ongoing hearing loss, speech and language difficulties, psychosocial difficulties, and lower educational attainment. (2–7) It is recommended that children with a cleft palate have surgery to repair their cleft when they are between 6 and 12 months old as this would reduce the likelihood of negative outcomes. (8,9) Cleft lip repair procedures are usually performed when the children are between 3 and 6 months old, a time frame suggested by a handful of small studies showing that early repair leads to better aesthetic results, (10,11) improved feeding,(10) and better psychosocial development. (12)

Access to healthcare declined markedly during the COVID-19 pandemic. (13,14) This decline represents both the postponement and cancellation of planned care. For some time-sensitive procedures such as cleft lip and palate repair, delays could have a detrimental effect on long-term outcomes.

This study aimed to quantify the impact of the COVID-19 pandemic on the number and the timing of first primary cleft lip and/or palate (CLP) repair procedures using national longitudinal administrative hospital data from the English National Health Service (NHS). We hypothesised that there would be a reduction in the number of first primary CLP repair procedures during the COVID-19 pandemic year (2020/21) compared to the preceding year (2019/20). We defined the start of the first COVID-19 pandemic year as 1st April 2020 as this coincides closely with the official start of the first nationwide lockdown in England on 23rd March 2020. (15)

For peer review only - http://bmjopen.bmj.com/site/about/guidelines.xhtml

We also hypothesised that first primary CLP repair procedures would be delayed during the pandemic, so that the children at the time of surgery would be older than in the preceding year. Quantifying the extent of delays to surgery is important for planning of the future needs of these children.

METHODS

Study design

Before/after study of the numbers of procedures, and age at surgery for primary repair of cleft lip and/or cleft palate in hospitals in England before (2019/20) or during (2020/21) the COVID-19 pandemic.

Data source and study population

We used the Hospital Episode Statistics (HES), a national database including records of all episodes in NHS hospitals derived from administrative data.(16) HES records include diagnostic fields coded according to the International Classification of Diseases – 10th revision (ICD-10) (17) and procedure fields coded according to the Population Consensus and Surveys Classification of Interventions and Procedures – 4th revisions (OPCS-4).(18)

We identified all children born after 1st April 2014, who were considered to have an orofacial cleft because they had both a record with relevant diagnostic codes before their second birthday (or until 31st March, 2021, whatever came earlier) and a record with relevant CLP repair procedure codes before their fifth birthday (or until 31st March, 2021, whatever came earlier; see supplementary information 1 for code lists). We excluded children without a birth record in HES and children born from multiple pregnancies. Births recorded in HES represent 97% of all births in England. (19) Please see Supplementary Figure 1.

Outcome and patient characteristics

In the children identified with an orofacial cleft, we determined the date of their first primary

For peer review only - http://bmjopen.bmj.com/site/about/guidelines.xhtml

CLP repair procedures with primary lip repair and primary palate repairs treated separately such that some children contributed more than one surgery. Secondary procedures were excluded from the analytical sample as other factors might influence their timing, including the timing of the primary surgery. We used diagnostic codes to distinguish four cleft types (see supplementary information 2 for code lists): cleft lip only (CL), cleft palate only (CP), unilateral cleft lip and palate (UCLP), bilateral cleft lip and palate (BCLP). We used procedure codes to capture the type of surgery: primary lip repair (F031) and primary palate repair (F291). We also used ICD-10 codes to determine whether there were other additional congenital malformations. (20,21) Quintiles of the national distribution of the 2019 Index of Multiple Deprivation (IMD) rankings of 32,844 Lower Super Output Areas (LSOA; areas with typically 1,500 inhabitants and 600 households) were used to categorise children into 5 groups according to their socioeconomic background. (22) Ethnicity was coded as White, and minority ethnicity including Black, Asian, mixed race, and other. Nine geographic regions of residence that correspond to the 9 regionally commissioned cleft services of England were derived from the LSOA.

Statistical analyses

We counted the number of first primary CLP repair procedures in 2020/21 and 2019/20 and calculated the relative difference between these numbers. Confidence intervals for these relative differences were calculated using the conditional method for testing differences between two Poisson means. (23) We used the Mantel-Haenszel test of homogeneity to investigate whether the difference between the number of procedures in 2019/20 and 2020/21 varied according to the children's characteristics.

To investigate changes in timing of first primary CLP repair procedures, we compared the mean age at the time of the first primary CLP procedures carried out in 2019/20 and 2020/21 with the t-test. Linear regression with interaction terms was used to test whether the

For peer review only - http://bmjopen.bmj.com/site/about/guidelines.xhtml

difference between the means in 2019/20 and 2020/21 varied according to the children's characteristics.

Children with missing data on a specific characteristic were not included in the analyses involving that characteristic. A p-value <0.05 was considered to indicate a statistically significant result. All analyses were performed in Stata V.17 (Statacorp). (24)

Patient and public involvement

The ECHILD project undertakes regular patient and public involvement (PPI) including the acceptability of the use of de-identified data from healthcare and education settings, and research priorities for these datasets. Children and parents in our PPI workshops identified understanding the health and education impact of the pandemic on children with additional clinical needs (such as CLP) as a key priority for research.

RESULTS

Study population

We identified 6,438 children with a CLP procedure code recorded before the age of 5 between 1st April 2014 and 31st March 2021. Of these, 680 (10.6%) did not have a birth record or were born from multiple pregnancies and 257 (4.0%) did not have a CLP diagnostic code recorded before the age of 2. These children were therefore excluded (Supplementary Figure 1).

<u>Number of first primary CLP repair procedures during the COVID-19 pandemic</u>

In the remaining 5,501 children, we identified 774 first primary CLP procedures in 2020/21 corresponding to 321 first lip repair and 453 first palate repairs. This was in comparison to 942 procedures (408 lip repairs, 534 palate repairs) in 2019/20, a reduction of 17.8 % (95% confidence interval 9.5% to 25.4%; p <0.001; Table 1).

For peer review only

Table 1: Number of first primary cleft lip and palate repair procedures by year of surgery and the children's characteristics

	**Number of procedures**							
	**Lip repair**				**Palate repairs**			
**Year of surgery***	**2019/20**	**2020/21**	**Relative difference (95% CI)**	**p-value**	**2019/20**	**2020/21**	**Relative difference (95% CI)**	**p-value**
**All**	408	321	-21.32 (-32.24, -8.71)	0.0013	534	453	-15.17 (-25.32, -3.67)	0.0099
**Cleft type**	**(p=0.6389) ****				**(p=0.8277) ****			
Cleft lip only	157	116	-26.11 (-42.39, -5.47)	0.0131	—	—	—	—
Cleft palate only	—	—	—	—	284	233	-17.96 (-31.31, -2.09)	0.0249
Unilateral CLP	165	141	-14.54 (-32.24, 7.65)	0.1707	164	147	-10.37 (-28.75, 12.67)	0.3358
Bilateral CLP	86	64	-25.58 (-47.01, 4.05)	0.073	86	73	-15.12 (-38.73, 17.32)	0.3041
**Congenital malformations**	**(p=0.1867) ****				**(p=0.0210) ****			
No additional malformations	282	207	-26.60 (-38.95, -11.86)	0.0007	240	237	-1.25 (-17.82, 18.66)	0.8909
Additional malformations	126	114	-9.52 (-30.39, 17.50)	0.4396	294	216	-26.53 (-38.65, -12.12)	0.0005
**IMD quintile**	**(p=0.9045) ****				**(p=0.8962) ****			
Q1 (Most deprived)	94	86	-8.51 (-32.51, 23.90)	0.5522	127	114	-10.24 (-30.91, 16.51)	0.4034
Q2	79	61	-22.78 (-45.64, 9.22)	0.1293	117	98	-16.24 (-36.62, 10.49)	0.196
Q3	65	53	-18.46 (-44.36, 19.02)	0.2712	92	78	-15.22 (-38.11, 15.90)	0.2843
Q4	61	45	-26.23 (-50.95, 10.23)	0.1215	62	51	-17.74 (-44.35, 21.11)	0.3029
Q5 (least deprived)	47	36	-23.40 (-51.79, 20.79)	0.2299	55	57	3.51 (-42.28, 34.61)	0.8509
Missing	62	40	—	—	81	55	—	—
**Ethnicity**	**(p=0.7415) ****				**(p=0.3669) ****			
White/White British	327	253	-22.63 (-34.60, -8.55)	0.0021	414	341	-17.63 (-28.84, -4.71)	0.0079
Minority ethnicity	74	61	-17.57 (-42.25, 17.28)	0.2649	112	106	-5.36 (-28.12, 24.55)	0.6852
Missing	**7**	**7**	—	—	8	6	—	—
**Region**	**(p=0.4821) ****				**(p=0.3715) ****			
North-East	25	20	-8.70 (-51.94, 72.56)	0.766	20	30	34.37 (-17.37, 64.03)	0.1337

For peer review only - http://bmjopen.bmj.com/site/about/guidelines.xhtml

North-West	45	50	6.12 (-43.34, 38.61)	0.7596	75	60	-18.42 (-42.63, 15.61)	0.2349
Yorkshire	40	30	-21.05 (-52.76, 30.85)	0.3356	55	40	-28.30 (-54.01, 10.82)	0.1174
East Midlands	35	30	-14.29 (-49.17, 43.71)	0.5386	35	25	-27.78 (-58.13, 22.98)	0.2074
West Midlands	30	30	-3.12 (-42.83, 63.95)	0.9007	45	50	9.80 (-37.07, 40.79)	0.6137
East of England	30	25	-28.12 (-59.84, 26.73)	0.2288	40	30	-28.57 (-56.83, 16.87)	0.1597
London	50	35	-28.57 (-55.07, 12.49)	0.1284	60	60	0 (-45.48, 31.26)	1.0000
South-East	50	45	-9.61 (-40.40, 36.74)	0.6173	65	65	-1.54 (-31.38, 41.24)	0.9302
South-West	35	15	-57.14 (-78.25, -19.47)	0.0046	40	30	-21.95 (-52.42, 27.01)	0.2954
Missing	65	40	—	—	95	60	—	—
**Quarter**	**(p<0.0001) ****				**(p<0.0001) ****			
Q1 (Apr-Jun)	105	10	-90.48 (-95.56, -81.78)	<0.0001	136	27	-80.15 (-87.38, -69.83)	<0.0001
Q2 (Jul-Sep)	107	110	2.73 (-26.16, 28.10)	0.839	149	182	18.13 (-2.22, 34.52)	0.07
Q3 (Oct-Dec)	103	140	26.43 (4.43, 43.52)	0.0177	123	130	5.38 (-22.01, 26.66)	0.6606
Q4 (Jan-Mar)	93	61	-34.41 (-53.31, -8.43)	0.0099	126	114	-9.52 (-30.39, 17.50)	0.4396

* 2020/21: first year of COVID-19 pandemic; 2019/20: preceding year.

** – Mantel-Haenszel test for homogeneity, testing if the relative differences vary according to the children's characteristics.

Region figures rounded to the nearest 5 for disclosure control.

For peer review only - http://bmjopen.bmj.com/site/about/guidelines.xhtml

The reduction in the number of first lip repair observed in 2020/21 did not vary significantly according to the children's characteristics (p always >0.1 for cleft type, presence of additional anomalies, deprivation or ethnicity) or geographic region of residence. However, the reduction in lip repairs did vary according to quarterly period (p<0.0001).

The reduction in the number of the first primary palate repair procedures in 2020/21 varied according to quarterly period (p<0.0001) and was significantly larger for children with additional congenital malformations (p=0.0210).

No repair procedures were carried out in the first two months of the study period (1st April to 31st May 2020), primary cleft surgery resumed in the third month of the first quarter. The numbers of first primary procedures undertaken in the second and third quarters of 2020/21 (1st July and 31st December 2020) were higher and the number in the fourth quarter (between 1st January to 31st March) was lower than in the corresponding months in the preceding year. (Figure 1)

Timing of CLP surgeries before and during the COVID-19 pandemic

The mean age at the first primary lip repairs increased by 1.6 months (95% CI: 0.9, 2.2) in the first year of the COVID-19 pandemic compared to 2019/20 (see also Figure 2). This increase in age did not vary according to the children's characteristics (p always > 0.1). The largest increases in mean age of lip repairs were in the South-West 3.9 months (95% CI:), the East Midlands 3.5 months (95% CI:), and the first quarter of 2020/2021 3.4 months (95% CI:). (Table 2)

For peer review only - http://bmjopen.bmj.com/site/about/guidelines.xhtml

Table 2: Mean age at first primary CLP repair surgery by year of surgery and exposure variables

	Mean age at surgery in months (95% CI)							
	Lip repair				Palate repairs			
**Year of surgery**	**2019/20**	**2020/21**	**Difference**	**p-value**	**2019/20**	**2020/21**	**Difference**	**p-value**
**All**	5.72 (5.28, 6.15)	7.30 (6.84, 7.76)	1.58 (0.94, 2.22)	<0.0001	11.19 (10.60, 11.77)	11.81 (11.30, 12.33)	0.63 (-0.16, 1.42)	0.1209
**Cleft type**	**(p=0.4166) ****				**(p=0.9728) ****			
Cleft lip only	5.42 (4.77, 6.08)	7.14 (6.22, 8.05)	1.71 (0.62, 2.80)	0.0022	—	—	—	—
Cleft palate only	—	—	—	—	12.74 (11.92, 13.57)	13.50 (12.74, 14.25)	0.75 (-0.39, 1.88)	0.1961
Unilateral CLP	5.46 (4.83, 6.10)	7.34 (6.81, 7.87)	1.88 (1.04, 2.72)	<0.0001	8.96 (8.10, 9.82)	9.66 (9.08, 10.25)	0.70 (-0.35, 1.76)	0.1919
Bilateral CLP	6.75 (5.58, 7.93)	7.51 (6.31, 8.70)	0.75 (-0.94, 2.45)	0.3816	10.28 (8.73, 11.83)	10.77 (9.29, 12.24)	0.49 (-1.66, 2.64)	0.6525
**Congenital malformations**	**(p=0.8086) ****				**(p=0.4014) ****			
No additional malformations	5.02 (4.68, 5.37)	6.56 (6.14, 6.98)	1.53 (0.99, 2.07)	<0.0001	9.27 (8.64, 9.90)	10.47 (9.95, 10.98)	1.20 (0.38, 2.01)	0.0039
Additional malformations	7.27 (6.13, 8.42)	8.65 (7.61, 9.68)	1.37 (-0.17, 2.91)	0.0813	12.75 (11.86, 13.65)	13.29 (12.41, 14.18)	0.54 (-0.75, 1.83)	0.4102
**IMD quintile**	**(p=0.9259) ****				**(p=0.9099) ****			
Q1 (Most deprived)	5.42 (4.58, 6.26)	7.29 (6.35, 8.23)	1.87 (0.62, 3.12)	0.0035	10.38 (9.29, 11.48)	11.64 (10.40, 12.88)	1.26 (-0.38, 2.90)	0.132
Q2	6.34 (5.09, 7.59)	8.25 (6.68, 9.82)	1.91 (-0.05, 3.87)	0.0561	10.87 (9.83, 11.91)	11.47 (10.54, 12.40)	0.60 (-0.82, 2.01)	0.4067
Q3	5.85 (5.02, 6.69)	7.18 (6.55, 7.81)	1.33 (0.25, 2.40)	0.0164	10.42 (9.29, 11.55)	11.51 (10.52, 12.50)	1.09 (-0.42, 2.61)	0.1557
Q4	5.96 (4.52, 7.41)	7.06 (5.77, 8.35)	1.10 (-0.89, 3.09)	0.2772	11.22 (9.63, 12.80)	12.45 (10.62, 14.28)	1.23 (-1.15, 3.62)	0.3086
Q5 (least deprived)	4.91 (4.40, 5.41)	6.83 (5.95, 7.72)	1.93 (0.98, 2.87)	0.0001	11.78 (10.21, 13.35)	13.68 (12.05, 15.32)	1.91 (-0.34, 4.15)	0.0949
**Ethnicity**	**(p=0.9406) ****				**(p=0.9348) ****			
White/White British	5.54 (5.09, 6.00)	7.23 (6.72, 7.75)	1.69 (1.00, 2.38)	<0.0001	11.34 (10.64, 12.04)	11.94 (11.34, 12.54)	0.60 (-0.34, 1.54)	0.2129
Minority ethnicity	6.00 (5.06, 6.93)	7.63 (6.44, 8.82)	1.63 (0.15, 3.11)	0.0312	10.81 (9.80, 11.82)	11.49 (10.40, 12.57)	0.68 (-0.80, 2.15)	0.3659
**Region**	**(p=0.2113) ****				**(p=0.0022) ****			
North-East	4.92 (3.44, 6.41)	5.51 (4.30, 6.72)	0.59 (-1.29, 2.47)	0.5297	10.27 (8.15, 12.39)	9.76 (8.39, 11.13)	-0.51 (-2.86, 1.83)	0.6622
North-West	5.10 (4.08, 6.12)	7.13 (5.80, 8.46)	2.03 (0.36, 3.70)	0.0176	8.45 (7.36, 9.54)	11.02 (9.15, 12.88)	2.56 (0.51, 4.62)	0.0146
Yorkshire	4.87 (4.08, 5.66)	7.00 (5.55, 8.45)	2.13 (0.59, 3.66)	0.0072	9.68 (8.37, 10.99)	11.21 (8.32, 14.10)	1.53 (-1.31, 4.37)	0.2881

For peer review only - http://bmjopen.bmj.com/site/about/guidelines.xhtml

East Midlands	4.71 (3.95, 5.46)	8.16 (6.81, 9.51)	3.45 (2.00, 4.91)	<0.0001	9.15 (7.56, 10.74)	11.36 (9.30, 13.42)	2.21 (-0.29, 4.72)	0.0827
West Midlands	6.41 (4.02, 8.80)	8.33 (7.59, 9.06)	1.91 (-0.57, 4.40)	0.1283	10.34 (8.60, 12.08)	11.20 (10.43, 11.96)	0.86 (-0.95, 2.67)	0.3492
East of England	5.55 (4.47, 6.64)	5.88 (5.11, 6.65)	0.33 (-1.08, 1.73)	0.6439	12.12 (9.60, 14.64)	12.14 (10.05, 14.22)	0.01 (-3.40, 3.43)	0.9935
London	6.36 (4.50, 8.22)	6.88 (4.56, 9.21)	0.52 (-2.38, 3.43)	0.7203	12.04 (10.09, 13.99)	10.83 (10.02, 11.64)	-1.21 (-3.30, 0.87)	0.252
South-East	6.51 (5.02, 8.00)	8.34 (6.84, 9.84)	1.83 (-0.26, 3.92)	0.0858	14.16 (12.34, 15.97)	13.91 (12.61, 15.21)	-0.24 (-2.46, 1.97)	0.829
South-West	5.83 (5.18, 6.48)	9.72 (8.50, 10.93)	3.89 (2.66, 5.12)	<0.0001	10.96 (9.59, 12.33)	16.51 (14.42, 18.60)	5.55 (3.18, 7.92)	<0.0001
**Quarter**	**(p=0.6010) ****				**(p=0.5800) ****			
Q1 (Apr-Jun)	5.52 (4.53, 6.51)	8.92 (7.15, 10.69)	3.40 (0.14, 6.66)	0.0412	10.79 (9.85, 11.74)	12.17 (11.11, 13.24)	1.38 (-0.80, 3.56)	0.2142
Q2 (Jul-Sep)	6.19 (5.18, 7.21)	7.47 (6.87, 8.07)	1.27 (0.11, 2.44)	0.0322	12.33 (11.01, 13.64)	12.34 (11.58, 13.09)	0.01 (-1.44, 1.45)	0.9915
Q3 (Oct-Dec)	5.39 (4.66, 6.12)	7.02 (6.22, 7.82)	1.63 (0.51, 2.75)	0.0046	10.62 (9.46, 11.77)	11.80 (10.54, 13.07)	1.19 (-0.52, 2.90)	0.1721
Q4 (Jan-Mar)	5.76 (5.08, 6.43)	7.37 (6.16, 8.57)	1.61 (0.34, 2.88)	0.0133	10.82 (9.62, 12.03)	10.91 (10.11, 11.71)	0.08 (-1.38, 1.55)	0.9093

* 2020/21: first year of COVID-19 pandemic; 2019/20: preceding year.

** Test for interaction testing to see if the differences in mean age vary according to the children's characteristic

For peer review only - http://bmjopen.bmj.com/site/about/guidelines.xhtml

At national level, mean age at the first primary palate repair did not increase during 2020/21 (0.6 months, 95% CI: -0.2, 1.4) but there was some evidence of regional variation (p=0.0022) with the largest increases in mean age being observed in the South-West (5.6 months; 95% CI: 3.2 to 7.9) and North-West (2.6 months; 95% CI: 0.5, 4.6).

There was an increase in the proportion of lip repairs carried out after the age of 6 months from 19.4% (79/408; 95% CI: 15.6%, 23.5%) in 2019/20 to 57.9% (186/321; 95% CI: 52.3%, 63.4%) in 2020/21 ($p < 0.0001$). There was also a small but significant increase in the proportion of palate repairs carried out after the age of 12 months from 22.5% (120/534; 95% CI: 19.0%, 26.2%) in 2019/20 to 28.7% (130/453; 95% CI: 24.6%, 33.1%) in 2020/21 ($p = 0.025$).

DISCUSSION

This national study using routinely collected administrative hospital data of children born with an orofacial cleft in England found an 18% reduction in the number of first primary CLP repair procedures during the first year of the COVID-19 pandemic, as well as a delay of 1.6 months in the timing of the first primary lip repair procedure, compared to the preceding year. The largest difference was observed during the first quarter of the COVID-19 pandemic period. Also, the delay in the timing of procedures varied across the country with children residing in the South-West most affected.

The study has several strengths. First, the study population had excellent geographical coverage of England, reflecting all NHS hospitals. Secondly, by using both diagnosis and procedure codes to identify the study population, the impact of coding errors on the differences reported will have been reduced. Third, the relatively large study population made it possible to report differences in number and timing of first primary CLP repair surgeries undertaken by patient characteristics, by region and quarterly period.

For peer review only - http://bmjopen.bmj.com/site/about/guidelines.xhtml

For peer review only

Limitations include that for some children data items on their specific diagnosis were missing and when differences were compared by the children's characteristics, our results were based only on a complete-case analysis. It is unclear what impact this may have on the results reported as it is not known whether children with missing data were more or less likely to have delayed surgery for CLP repair than those with complete data. The use of ICD-10 and OPCS-4 codes may not capture more nuanced clinical information about individual diagnoses and procedures.

We showed that CLP repair surgery completely stopped in April and May 2020, which coincides with the start of the first national "lockdown" in England on 26th March 2020. This translated to a reduction in numbers of both primary lip repairs and primary palate repairs. Stakeholders will need to continue monitoring this as these reductions could have long term consequences (e.g. on speech development) and may have a time lag in their effect. The reduction in the number of first palate repair surgeries for children with additional congenital malformations was larger than for children without additional malformations, which may reflect deferred surgeries for children at higher risk of complications from COVID-19 (25). We also showed an increase in the age at first lip repair surgery, but no significant increase in the age at first palate repair. This may reflect clinical prioritisation of primary cleft palate repairs over cleft lip repairs. UK national guidance suggests that palate repair should be complete by 13 months of age (guidance palate repair at age 6 to 13 months) (UK guidance; 3-6 months of age for lip repairs)(26,27). However, we also showed that a significantly larger proportion of children had their first palate repair surgery after 12 months which might have long term consequences for education attainment as children who receive palate repairs after 13 months have been shown to have less favourable speech outcomes. (8,9)

Our study indicated that there were regional variations in the impact of the COVID-19 pandemic on the timing of first primary CLP repair procedures, which may reflect differences

For peer review only - http://bmjopen.bmj.com/site/about/guidelines.xhtml

in the regions' influence on management decision making, resources, fragility, and capacity for recovery. The delay of almost 6 months seen in one region with other regions showing hardly any delay in the timing of first primary CLP repair procedures requires further investigation.

This paper follows on from previous work which showed reductions in planned care during the pandemic and acts as a deeper dive into one specific type of planned care. (28) This work focuses on primary procedures which were given some prioritisation during the pandemic and as such might downplay its effect on wider cleft services. For example, secondary procedures such as Alveolar bone graft and secondary speech surgery are time sensitive but have less evidence supporting them. While the Federation of Specialist Surgical Associations Clinical Guide to Surgical Prioritisation during the COVID pandemic gave similar priority to primary and secondary cleft procedures, shop floor practicality may not necessarily have allowed equal treatment. (29) Further work needs to be done to understand the full effect of the pandemic on all cleft surgery especially the more temporarily sensitive secondary cleft procedures (alveolar bone grafting and secondary speech surgery).

In conclusion, during the first year of the COVID-19 pandemic a larger proportion of children had their cleft repair surgery outside of the recommended timeframe (3 to 6 months for lip repair and 6 to 13 months for palate repair). Previous research has shown that late surgery may be associated with delays in speech development and the need for additional speech therapy.(8,9) Delayed surgery beyond 13 months is thought to affect articulation following cleft palate repair, and the resulting need for extra corrective speech therapy may contribute to additional absence from school, potentially affecting primary educational attainment.(7,30) Future research should therefore consider investigating the effect of delay in surgery on educational outcomes to model the long-term implications of the COVID pandemic.

For peer review only - http://bmjopen.bmj.com/site/about/guidelines.xhtml

FUNDING:

This project was funded by the National Institute for Health and Care Research (NIHR) Policy Research Programme. The views expressed are those of the author(s) and not necessarily those of the NIHR or the Department of Health and Social Care. This research was supported in part by the NIHR Great Ormond Street Hospital Biomedical Research Centre and the Health Data Research UK (grant no: LOND1), which is funded by the UK Medical Research Council and eight other funders. RB is supported by a UKRI Innovation Fellowship funded by the Medical Research Council (grant number MR/S003797/1).

AUTHOR CONTRIBUTIONS

JVDM and MHP developed the research question. DE, LMGL and RB operationalised the research question jointly with JVDM and MHP using data available via UCL Child Health Informatics team. DE undertook the analysis and drafted the manuscript. KF, SB, JM and CR provided essential clinical and contextual input into the study design and analysis, and interpretation of the results. All authors provided oversight and input to the final manuscript.

ACKNOWLEDGEMENTS

We thank Professor Ruth Gilbert and Professor Katie Harron for their support of the ECHILD project.

What is already known on this topic:

For peer review only

- Surgical repair of cleft lip and palate (CLP) should promote optimal outcomes including feeding, speech and aesthetics, with enduring consequences for child development and education.
- Clinical guidelines advocate surgery for first repair of cleft lip in children aged 3-6 months and before age 13 months for cleft palate repair.
- The COVID-19 pandemic significantly disrupted planned healthcare, but the impact on the timing of surgery for primary repair of CLP is not known.

What this study adds:

- During the pandemic, over half of children with cleft lip and over one quarter with cleft palate exceeded the recommended age for first primary repair.
- Overall, the number of procedures for primary CLP repair during the first year of the COVID-19 pandemic was 18% lower than the prior year.
- First primary lip repair was delayed by an average of 1.6 months, with no evidence of a delay in primary palate repair at national level.

How this study might affect research, practice or policy:

- Services for children with CLP should be aware that targeted support may be required to mitigate the longer-term effects of surgical delays during the pandemic.
- Determining the impact of delays in primary CLP repair on child development and education outcomes is a research priority.
- Such research is needed both to support children affected by the pandemic and to inform the evidence-base for the optimal timing of CLP surgery.

ETHICAL APPROVAL

For peer review only - http://bmjopen.bmj.com/site/about/guidelines.xhtml

Ethical approval was not required for this study because only de-identified routinely collected data were analysed.

CONFLICTS OF INTEREST

The authors have no conflicts of interest to declare

DATA SHARING STATEMENT

Data are available on request from NHS Digital and may not be shared by the authors.

REFERENCES

1. Cleft Lip and Palate Association (CLAPA), Cleft Registry and Audit Network (CRANE). CRANE 2020 Annual Report: Summary of findings for patients and parents/carers on children born with a cleft in England, Wales and Northern Ireland between January 2000 and December 2019. [Internet]. 2021 [cited 2022 Feb 3]. Available from: https://www.crane-database.org.uk/content/uploads/2021/03/CRANE-2020-AR_Patients-Parents-Carers-Summary_V1.pdf

2. Fitzsimons KJ, Copley LP, Van Der Meulen JH, Panagamuwa C, Deacon SA. Grommet Surgery in Children with Orofacial Clefts in England. Cleft Palate Craniofac J. 2017 Jan 1;54(1):80–9. https://doi.org/10.1597/15-047

3. Mildinhall S. Speech and Language in the Patient with Cleft Palate. Cleft Lip Palate. 2012;16:137–46. https://doi.org/10.1159/000337668

4. Stock N, Rumsey N. Living with a Cleft: Psychological Challenges, Support and Intervention. In: Cleft Lip and Palate: Diagnosis and Management. 2013. https://doi.org/10.1007/978-3-642-30770-6_45

For peer review only

For peer review only - http://bmjopen.bmj.com/site/about/guidelines.xhtml

5. Gallagher ER, Collett BR. Neurodevelopmental and Academic Outcomes in Children With Orofacial Clefts: A Systematic Review. Pediatrics. 2019 Jul 1;144(1):e20184027. https://doi.org/10.1542/peds.2018-4027

6. Fitzsimons KJ, Copley LP, Setakis E, Charman SC, Deacon SA, Dearden L, et al. Early academic achievement in children with isolated clefts: a population-based study in England. Arch Dis Child. 2018 Apr 1;103(4):356–62. https://doi.org/10.1136/archdischild-2017-313777

7. Fitzsimons KJ, Deacon SA, Copley LP, Park MH, Medina J, van der Meulen JH. School absence and achievement in children with isolated orofacial clefts. Arch Dis Child. 2021 Feb 1;106(2):154–9. https://doi.org/10.1136/archdischild-2020-319123

8. Shaffer AD, Ford MD, Losee JE, Goldstein J, Costello BJ, Grunwaldt LJ, et al. The Association Between Age at Palatoplasty and Speech and Language Outcomes in Children With Cleft Palate: An Observational Chart Review Study. Cleft Palate Craniofac J. 2020 Feb 1;57(2):148–60. https://doi.org/10.1177/1055665619882566

9. Lindeborg MM, Shakya P, Rai SM, Shaye DA. Optimizing speech outcomes for cleft palate. Curr Opin Otolaryngol Head Neck Surg. 2020 Aug;28(4):206–11. https://doi.org/10.1097/MOO.0000000000000635

10. Jiri B, Jana V, Michal J, Jiri K, Dana H, Miroslav T, et al. Successful early neonatal repair of cleft lip within first 8 days of life. Int J Pediatr Otorhinolaryngol. 2012 Nov 1;76(11):1616–26. https://doi.org/10.1016/j.ijporl.2012.07.031

11. Hammoudeh JA, Imahiyerobo TA, Liang F, Fahradyan A, Urbinelli L, Lau J, et al. Early Cleft Lip Repair Revisited: A Safe and Effective Approach Utilizing a Multidisciplinary Protocol. Plast Reconstr Surg – Glob Open. 2017 Jun;5(6):e1340. https://doi.org/10.1097/GOX.0000000000001340

For peer review only

For peer review only - http://bmjopen.bmj.com/site/about/guidelines.xhtml

12. Petráčková I, Zach J, Borský J, Černý M, Hacklová R, Tvrdek M, et al. Early and late operation of cleft lip and intelligence quotient and psychosocial development in 3–7years. Early Hum Dev. 2015 Feb 1;91(2):149–52. https://doi.org/10.1016/j.earlhumdev.2014.12.015

13. Isba R, Edge R, Jenner R, Broughton E, Francis N, Butler J. Where have all the children gone? Decreases in paediatric emergency department attendances at the start of the COVID-19 pandemic of 2020. Arch Dis Child. 2020 Jul 1;105(7):704–704. https://doi.org/10.1136/archdischild-2020-319385

14. Bottle A, Aylin P, Warner M, Propper C, Stoye G, Burn S. What happened to English NHS hospital activity during the COVID-19 pandemic? [Internet]. 2021 May [cited 2021 Nov 30]. Available from: https://ifs.org.uk/publications/15432

15. Timeline of UK government coronavirus lockdowns and restrictions [Internet]. The Institute for Government. 2021 [cited 2022 Sep 5]. Available from: https://www.instituteforgovernment.org.uk/charts/uk-government-coronavirus-lockdowns

16. Herbert A, Wijlaars L, Zylbersztejn A, Cromwell D, Hardelid P. Data Resource Profile: Hospital Episode Statistics Admitted Patient Care (HES APC). Int J Epidemiol. 2017 Aug 1;46(4):1093–1093i. https://doi.org/10.1093/ije/dyx015

17. WHO | ICD-10 online versions [Internet]. WHO. World Health Organization; [cited 2020 Apr 20]. Available from: http://www.who.int/classifications/icd/icdonlineversions/en/

18. OPCS classification of interventions and procedures. Version 4.8. Version 4.8 (2017). London: Stationery Office; 2017. Available from: https://digital.nhs.uk/data-and-information/information-standards/information-standards-and-data-collections-including-extractions/publications-and-notifications/standards-and-collections/dapb0084-opcs-classification-of-interventions-and-procedures

For peer review only - http://bmjopen.bmj.com/site/about/guidelines.xhtml

19. Harron K, Gilbert R, Cromwell D, Meulen J van der. Linking Data for Mothers and Babies in De-Identified Electronic Health Data. PLOS ONE. 2016 Oct 20;11(10):e0164667. https://doi.org/10.1371/journal.pone.0164667

20. Fitzsimons K, Hamilton M, van der Meulen J, Medina J, Wahedally M, Park M, et al. Range and Frequency of Congenital Malformations Among Children With Cleft Lip and/or Palate. Cleft Palate Craniofac J. 2022 Apr 5;10556656221089160. https://doi.org/10.1177/10556656221089160

21. Zylbersztejn A, Verfürden M, Hardelid P, Gilbert R, Wijlaars L. Phenotyping congenital anomalies in administrative hospital records. Paediatr Perinat Epidemiol. 2020 Jan;34(1):21–8. https://doi.org/10.1111/ppe.12627

22. Office of the Deputy Prime Minister, Neighbourhood Renewal Unit. The English indices of deprivation 2004. [Internet]. London: Office of the Deputy Prime Minister; 2004. Available from: https://www.brent.gov.uk/media/323946/SD%2062b%20Indices%20of%20multiple%20deprivation%202004.pdf

23. Hilbe JM. Modeling Count Data [Internet]. Cambridge: Cambridge University Press; 2014 [cited 2022 Sep 5]. Available from: https://www.cambridge.org/core/books/modeling-count-data/BFEB3985905CA70523D9F98DA8E64D08

24. StataCorp. Stata Statistical Software. College Station, TX: StataCorp LLC; 2017.

25. COVID-19: guidance on protecting people defined on medical grounds as extremely vulnerable [Internet]. GOV.UK. [cited 2022 Dec 16]. Available from: https://www.gov.uk/government/publications/guidance-on-shielding-and-protecting-extremely-vulnerable-persons-from-covid-19

For peer review only

For peer review only - http://bmjopen.bmj.com/site/about/guidelines.xhtml

26. Federation of Surgical Specialty Association. Clinical Guide to Surgical Prioritisation During the Coronavirus Pandemic [Internet]. [cited 2022 Sep 5]. Available from: https://fssa.org.uk/_userfiles/pages/files/covid19/prioritisation_master_250920.pdf

27. Resumption of Cleft Services [Internet]. CLAPA. [cited 2022 Feb 3]. Available from: https://www.clapa.com/news-item/resumption-of-cleft-services/

28. Etoori D, Harron KL, Grath-Lone LM, Verfürden ML, Gilbert R, Blackburn R. Reductions in hospital care among clinically vulnerable children aged 0–4 years during the COVID-19 pandemic. Arch Dis Child [Epub ahead of print]. 2022 Jun 20 [cited 2022 Sep 5]; https://doi.org/10.1136/archdischild-2021-323681

29. Clinical Guide to Surgical Prioritisation During the Coronavirus Pandemic [Internet]. FSSA. [cited 2022 Dec 15]. Available from: https://fssa.org.uk/covid-19_documents.aspx

30. Russell CJH, Fitzsimons KJ, Britton L, Van Eeden S, Butterworth S, Wahedally MHA, Park MH, Medina J, van der Meulen J, What Is The Optimal Timing Of Palate Repair For Speech Outcomes? Analysis Of Two Linked National Databases In England. [Oral presentation] CLEFT 2022 conference

For peer review only - http://bmjopen.bmj.com/site/about/guidelines.xhtml

Figure 1: Monthly numbers of first primary cleft lip repair and palate repair procedures in the first year of the COVID-19 period (between April 2020 and March 2021; red line) and the preceding year (between April 2019 and March 2020; blue line).

* Grey lines represent 5-year average (14/15 to 18/19) for historic comparison. Shaded areas represent lockdown periods (Lockdown 1: March 23 – June 23, 2020; Lockdown 2: November 5 – December 6, 2020; Lockdown 3: January 1 – March 8, 2021). CLP – Cleft lip and palate

For peer review only

Figure 2: Mean age at the first primary cleft lip and palate repair procedure in the first year of the COVID-19 pandemic (between April 2020 and March 2021; red square) and the preceding year (between April 2019 and March 2020; blue circle) with 95% confidence intervals.

Q1 – April – June; Q2 – July – September; Q3 – October – December; Q4 – January – March.

For peer review only

For peer review only - http://bmjopen.bmj.com/site/about/guidelines.xhtml

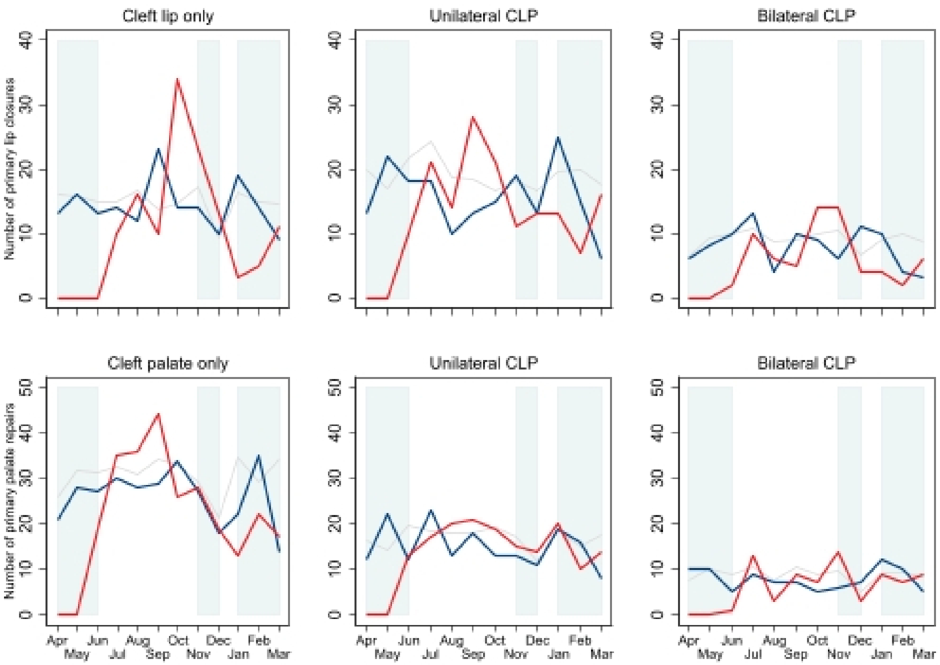

44x32mm (300 x 300 DPI)

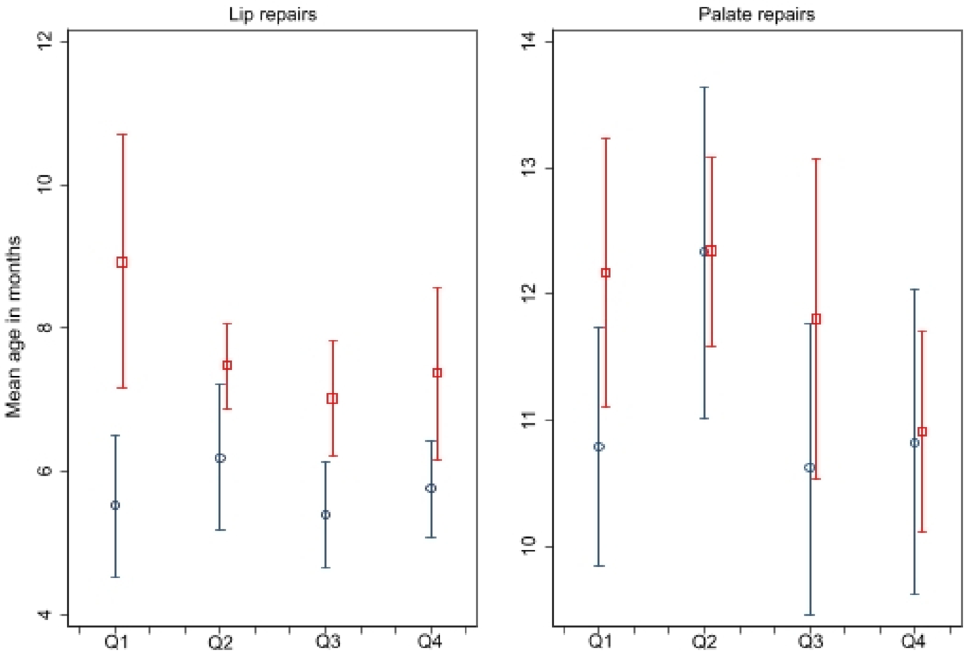

44x32mm (300 x 300 DPI)

SUPPLEMENTARY MATERIALS

Supplementary Figure 1: Flow chart showing inclusion criteria into the study

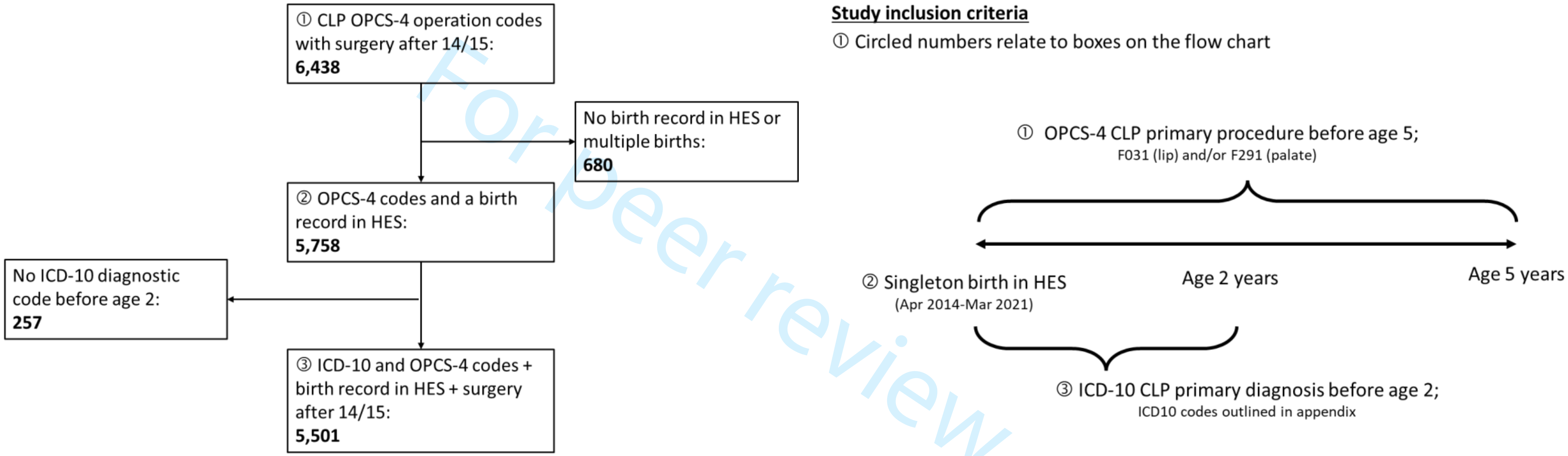

For peer review only

For peer review only - http://bmjopen.bmj.com/site/about/guidelines.xhtml

Supplementary information 1: OPCS-4 CLP surgery procedure code lists

OPCS-4 code	Surgery type
F031	Primary closure of cleft lip
F291	Primary palate repair

Supplementary information 2: Cleft lip and palate ICD-10 codes

ICD-10 codes	Cleft type
Q35x	Cleft lip
Q36x	Cleft palate
Q371, Q373, Q375, Q379	Unilateral cleft lip and palate
Q370, Q372, Q374, Q378	Bilateral cleft lip and palate

For peer review only - http://bmjopen.bmj.com/site/about/guidelines.xhtml

Supplementary information 3: Congenital anomalies code list

Code	Description
	Congenital malformations of the nervous system
Q00	Anencephaly and similar malformations
Q01	Encephalocele
Q02	Microcephaly
Q03	Congenital hydrocephalus
Q04	Other congenital malformations of brain
Q05	Spina bifida
Q06	Other congenital malformations of spinal cord
Q07	Other congenital malformations of nervous system
	Congenital malformations of eye, ear, face and neck
Q10	Congenital ptosis
Q11	Anophthalmos, microphthalmos and macrophthalmos
Q12	Congenital lens malformations
Q13	Congenital malformations of anterior segment of eye
Q14	Congenital malformations of posterior segment of eye
Q15	Other congenital malformations of eye
Q16	Congenital malformations of ear causing impairment of hearing
Q17	Other congenital malformations of ear

For peer review only - http://bmjopen.bmj.com/site/about/guidelines.xhtml

Q18	Other congenital malformations of face and neck
	Congenital malformations of the circulatory system
Q20	Congenital malformations of cardiac chambers and connections
Q21	Congenital malformations of cardiac septa
Q22	Congenital malformations of pulmonary and tricuspid valves
Q23	Congenital malformations of aortic and mitral valves
Q24	Other congenital malformations of heart
Q25	Congenital malformations of great arteries
Q26	Congenital malformations of great veins
Q27	Other congenital malformations of peripheral vascular system
Q28	Other congenital malformations of circulatory system
	Congenital malformations of the respiratory system
Q30	Congenital malformations of nose
Q31	Congenital malformations of larynx
Q32	Congenital malformations of trachea and bronchus
Q33	Congenital malformations of lung
Q34	Other congenital malformations of respiratory system
	Other congenital malformations of the digestive system
Q38	Other congenital malformations of tongue, mouth and pharynx
Q39	Congenital malformations of oesophagus
Q40	Other congenital malformations of upper alimentary tract
Q41	Congenital absence, atresia and stenosis of small intestine

For peer review only

Q42	Congenital absence, atresia and stenosis of large intestine
Q43	Other congenital malformations of intestine
Q44	Congenital malformations of gallbladder, bile ducts and liver
Q45	Other congenital malformations of digestive system
	Congenital malformations of the genital organs
Q50	Congenital malformations of ovaries, fallopian tubes and broad ligaments
Q51	Congenital malformations of uterus and cervix
Q52	Other congenital malformations of female genitalia
Q53	Undescended testicle
Q54	Hypospadias
Q55	Other congenital malformations of male genital organs
Q56	Indeterminate sex and pseudohermaphroditism
	Congenital malformations of the urinary system
Q60	Renal agenesis and other reduction defects of kidney
Q61	Cystic kidney disease
Q62	Congenital obstructive defects of renal pelvis and congenital malformations of ureter
Q63	Other congenital malformations of kidney
Q64	Other congenital malformations of urinary system
	Congenital malformations and deformations of the musculoskeletal system
Q65	Congenital deformities of hip
Q66	Congenital deformities of feet
Q67	Congenital musculoskeletal deformities of head, face, spine and chest

For peer review only

For peer review only - http://bmjopen.bmj.com/site/about/guidelines.xhtml

Q68	Other congenital musculoskeletal deformities
Q69	Polydactyly
Q70	Syndactyly
Q71	Reduction defects of upper limb
Q72	Reduction defects of lower limb
Q73	Reduction defects of unspecified limb
Q74	Other congenital malformations of limb(s)
Q75	Other congenital malformations of skull and face bones
Q76	Congenital malformations of spine and bony thorax
Q77	Osteochondrodysplasia with defects of growth of tubular bones and spine
Q78	Other osteochondrodysplasias
Q79	Congenital malformations of the musculoskeletal system, not elsewhere classified
	Other congenital malformations
Q80	Congenital ichthyosis
Q81	Epidermolysis bullosa
Q82	Other congenital malformations of skin
Q83	Congenital malformations of breast
Q84	Other congenital malformations of integument
Q85	Phakomatoses, not elsewhere classified
Q86	Congenital malformation syndromes due to known exogenous causes, not elsewhere classified
Q87	Other specified congenital malformation syndromes affecting multiple systems
Q89	Other congenital malformations, not elsewhere classified

For peer review only - http://bmjopen.bmj.com/site/about/guidelines.xhtml

For peer review only

	Chromosomal abnormalities, not elsewhere classified
Q90	Down syndrome
Q91	Edwards syndrome and Patau syndrome
Q92	Other trisomies and partial trisomies of the autosomes, not elsewhere classified
Q93	Monosomies and deletions from the autosomes, not elsewhere classified
Q95	Balanced rearrangements and structural markers, not elsewhere classified
Q96	Turner syndrome
Q97	Other sex chromosome abnormalities, female phenotype, not elsewhere classified
Q98	Other sex chromosome abnormalities, male phenotype, not elsewhere classified
Q99	Other chromosome abnormalities, not elsewhere classified

For peer review only

For peer review only - http://bmjopen.bmj.com/site/about/guidelines.xhtml

STROBE Statement—checklist of items that should be included in reports of observational studies

	Item No	Recommendation	Page No
**Title and abstract**	1	(*a*) Indicate the study's design with a commonly used term in the title or the abstract	1
		(*b*) Provide in the abstract an informative and balanced summary of what was done and what was found	2-3
**Introduction**			
Background/rationale	2	Explain the scientific background and rationale for the investigation being reported	3-4
Objectives	3	State specific objectives, including any prespecified hypotheses	4
**Methods**			
Study design	4	Present key elements of study design early in the paper	5
Setting	5	Describe the setting, locations, and relevant dates, including periods of recruitment, exposure, follow-up, and data collection	5-6
Participants	6	(*a*) *Cohort study*—Give the eligibility criteria, and the sources and methods of selection of participants. Describe methods of follow-up<br>*Case-control study*—Give the eligibility criteria, and the sources and methods of case ascertainment and control selection. Give the rationale for the choice of cases and controls<br>*Cross-sectional study*—Give the eligibility criteria, and the sources and methods of selection of participants	5-6
		(*b*) *Cohort study*—For matched studies, give matching criteria and number of exposed and unexposed<br>*Case-control study*—For matched studies, give matching criteria and the number of controls per case	
Variables	7	Clearly define all outcomes, exposures, predictors, potential confounders, and effect modifiers. Give diagnostic criteria, if applicable	5-6
Data sources/ measurement	8*	For each variable of interest, give sources of data and details of methods of assessment (measurement). Describe comparability of assessment methods if there is more than one group	4-5
Bias	9	Describe any efforts to address potential sources of bias	5
Study size	10	Explain how the study size was arrived at	5
Quantitative variables	11	Explain how quantitative variables were handled in the analyses. If applicable, describe which groupings were chosen and why	6
Statistical methods	12	(*a*) Describe all statistical methods, including those used to control for confounding	6
		(*b*) Describe any methods used to examine subgroups and interactions	6
		(*c*) Explain how missing data were addressed	7
		(*d*) *Cohort study*—If applicable, explain how loss to follow-up was addressed<br>*Case-control study*—If applicable, explain how matching of cases and controls was addressed<br>*Cross-sectional study*—If applicable, describe analytical methods taking account of sampling strategy	6
		(*e*) Describe any sensitivity analyses	

Continued on next page

For peer review only - http://bmjopen.bmj.com/site/about/guidelines.xhtml

**Results**			
Participants	13*	(a) Report numbers of individuals at each stage of study—eg numbers potentially eligible, examined for eligibility, confirmed eligible, included in the study, completing follow-up, and analysed	7 & Suppl Fig 1
		(b) Give reasons for non-participation at each stage	
		(c) Consider use of a flow diagram	
Descriptive data	14*	(a) Give characteristics of study participants (eg demographic, clinical, social) and information on exposures and potential confounders	Table 1
		(b) Indicate number of participants with missing data for each variable of interest	
		(c) *Cohort study*—Summarise follow-up time (eg, average and total amount)	
Outcome data	15*	*Cohort study*—Report numbers of outcome events or summary measures over time	
		Case-control study—Report numbers in each exposure category, or summary measures of exposure	
		Cross-sectional study—Report numbers of outcome events or summary measures	7, T1
Main results	16	(*a*) Give unadjusted estimates and, if applicable, confounder-adjusted estimates and their precision (eg, 95% confidence interval). Make clear which confounders were adjusted for and why they were included	7-10
		(*b*) Report category boundaries when continuous variables were categorized	7-10
		(*c*) If relevant, consider translating estimates of relative risk into absolute risk for a meaningful time period	
Other analyses	17	Report other analyses done—eg analyses of subgroups and interactions, and sensitivity analyses	7-10
**Discussion**			
Key results	18	Summarise key results with reference to study objectives	14
Limitations	19	Discuss limitations of the study, taking into account sources of potential bias or imprecision. Discuss both direction and magnitude of any potential bias	15
Interpretation	20	Give a cautious overall interpretation of results considering objectives, limitations, multiplicity of analyses, results from similar studies, and other relevant evidence	15-16
Generalisability	21	Discuss the generalisability (external validity) of the study results	14
**Other information**			
Funding	22	Give the source of funding and the role of the funders for the present study and, if applicable, for the original study on which the present article is based	17

*Give information separately for cases and controls in case-control studies and, if applicable, for exposed and unexposed groups in cohort and cross-sectional studies.

Note: An Explanation and Elaboration article discusses each checklist item and gives methodological background and published examples of transparent reporting. The STROBE checklist is best used in conjunction with this article (freely available on the Web sites of PLoS Medicine at http://www.plosmedicine.org/, Annals of Internal Medicine at http://www.annals.org/, and Epidemiology at http://www.epidem.com/). Information on the STROBE Initiative is available at www.strobe-statement.org.

For peer review only - http://bmjopen.bmj.com/site/about/guidelines.xhtml

BMJ Open

Number and timing of primary cleft lip and palate repair surgeries in England: whole nation study of electronic health records before and during the COVID-19 pandemic.

Journal:	*BMJ Open*
Manuscript ID	bmjopen-2023-071973.R1
Article Type:	Original research
Date Submitted by the Author:	20-Apr-2023
Complete List of Authors:	Etoori, David; University College London Institute of Health Informatics<br>Park, Min Hae; London School of Hygiene & Tropical Medicine<br>Blackburn, Ruth; University College London Institute of Health Informatics<br>Fitzsimons, Kate; Royal College of Surgeons of England<br>Butterworth, Sophie; Royal College of Surgeons of England<br>Medina, Jibby; Royal College of Surgeons of England, Clinical Effectiveness Unit<br>Mc Grath-Lone, Louise; University College London Institute of Education<br>Russell, Craig; Queen Elizabeth University Hospital<br>van der Meulen, Jan; London School of Hygiene & Tropical Medicine
**Primary Subject Heading**:	Surgery
Secondary Subject Heading:	Epidemiology
Keywords:	COVID-19, Paediatric surgery < SURGERY, EPIDEMIOLOGY, PAEDIATRIC SURGERY

SCHOLARONE™
Manuscripts

I, the Submitting Author has the right to grant and does grant on behalf of all authors of the Work (as defined in the below author licence), an exclusive licence and/or a non-exclusive licence for contributions from authors who are: i) UK Crown employees; ii) where BMJ has agreed a CC-BY licence shall apply, and/or iii) in accordance with the terms applicable for US Federal Government officers or employees acting as part of their official duties; on a worldwide, perpetual, irrevocable, royalty-free basis to BMJ Publishing Group Ltd ("BMJ") its licensees and where the relevant Journal is co-owned by BMJ to the co-owners of the Journal, to publish the Work in this journal and any other BMJ products and to exploit all rights, as set out in our licence.

The Submitting Author accepts and understands that any supply made under these terms is made by BMJ to the Submitting Author unless you are acting as an employee on behalf of your employer or a postgraduate student of an affiliated institution which is paying any applicable article publishing charge ("APC") for Open Access articles. Where the Submitting Author wishes to make the Work available on an Open Access basis (and intends to pay the relevant APC), the terms of reuse of such Open Access shall be governed by a Creative Commons licence – details of these licences and which Creative Commons licence will apply to this Work are set out in our licence referred to above.

Other than as permitted in any relevant BMJ Author's Self Archiving Policies, I confirm this Work has not been accepted for publication elsewhere, is not being considered for publication elsewhere and does not duplicate material already published. I confirm all authors consent to publication of this Work and authorise the granting of this licence.

For peer review only

Title: Number and timing of primary cleft lip and palate repair surgeries in England: whole nation study of electronic health records before and during the COVID-19 pandemic.

Authors: David Etoori[1], Min Hae Park[2], Ruth Blackburn[1*], Kate Fitzsimons,[3] Sophie Butterworth,[3] Jibby Medina[3], Louise Mc Grath-Lone[1], Craig Russell[4], Jan Van Der Meulen[2]

Affiliations:

1. University College London Institute of Health Informatics, 222 Euston Road, London, NW1 2DA, UK.
2. London School of Hygiene and Tropical Medicine, Keppel Street, London, WC1E 7HT, UK.
3. Clinical Effectiveness Unit, Royal College of Surgeons of England, 38 – 43 Lincoln's Inn Fields, London, UK.
4. Royal Hospital for Children, Queen Elizabeth University Hospital, Glasgow, UK

*** Corresponding author:**

Dr Ruth Blackburn, 222 Euston Road, London, NW1 2DA, UK, Email: r.blackburn@ucl.ac.uk

Email addresses: DE: d.etoori@ucl.ac.uk**;** MHP: MinHae.Park@lshtm.ac.uk; RB: r.blackburn@ucl.ac.uk; KF: kfitzsimons@rcseng.ac.uk**;** SB: sbutterworth@rcseng.ac.uk**;** JM; jmedina@rcseng.ac.uk; LMcGL: l.mcgrath-lone@ucl.ac.uk**;** CR: craig.russell@ggc.scot.nhs.uk; JVDM: Jan.vanderMeulen@lshtm.ac.uk

Key words: COVID-19; orofacial cleft, cleft lip; cleft palate; timing of surgery

For peer review only

For peer review only - http://bmjopen.bmj.com/site/about/guidelines.xhtml

Word count: Abstract 227; Text 2531

ABSTRACT

Objective To quantify differences in number and timing of first primary cleft lip and palate (CLP) repair procedures during the first year of the COVID-19 pandemic (1st April 2020 to 31st March 2021; 2020/21) compared to the preceding year (1st April 2019 to 31st March 2020; 2019/21).

Design National observational study of administrative hospital data.

Setting National Health Service hospitals in England.

Study population Children <5 years undergoing primary repair for an orofacial cleft (OPCS-4 codes F031, F291)

Main exposure Procedure date (2020/21 vs 2019/20)

Main outcomes Numbers and timing (age in months) of first primary CLP procedures.

Results 1,716 CLP primary repair procedures were included in the analysis. In 2020/21, 774 CLP procedures were carried out compared to 942 in 2019/20, a reduction of 17.8 % (95% confidence interval 9.5% to 25.4%). The reduction varied over time in 2020/21, with no surgeries at all during the first two months (April and May 2020). Compared to 2019/20, first primary lip repair procedures performed in 2020/21 were delayed by 1.6 months on average (95% confidence interval 0.9 to 2.2 months). Delays in primary palate repairs were smaller on average but varied across the nine geographical regions.

Conclusion There were significant reductions in the number and delays in timing of first primary CLP repair procedures in England during the first year of the pandemic, which may affect long-term outcomes.

For peer review only

STRENGTHS AND LIMITATIONS OF THIS STUDY

- We analysed administrative hospital data (Hospital Episodes Statistics; HES) with whole nation coverage of England for children undergoing surgical repair of cleft lip and palate (CLP) at in two time periods; before (2019/20) or during (2020/21) the COVID-19 pandemic.
- Within these time periods we examined the timing of first surgical repair with respect to clinical guidelines advocating surgery for first repair of cleft lip in children aged 3-6 months and before age 13 months for cleft palate repair
- To reduce the risk of misclassifying the timing of surgery we restricted the study population to children born in hospitals in England, meaning that some children who had CLP surgery (but who did not have a birth record in HES) were excluded from the analysis.
- Even though our study had whole nation coverage of England the numbers of children within some important sub-groups (e.g. narrower ethnic groups) were insufficient to support further analysis.

For peer review only

INTRODUCTION

Around 1 in 670 children in the England, Wales and Northern Ireland are born alive with an orofacial cleft that may affect only the lip, only the palate, or both. (1) An orofacial cleft can have significant effects on children's lives, including ongoing hearing loss, speech and language difficulties, psychosocial difficulties, and lower educational attainment. (2–7) It is recommended that children with a cleft palate have surgery to repair their cleft when they are between 6 and 12 months old as this would reduce the likelihood of negative outcomes. (8,9)

For peer review only - http://bmjopen.bmj.com/site/about/guidelines.xhtml

Cleft lip repair procedures are usually performed when the children are between 3 and 6 months old, a time frame suggested by a handful of small studies showing that early repair leads to better aesthetic results, (10,11) improved feeding,(10) and better psychosocial development. (12)

Access to healthcare declined markedly during the COVID-19 pandemic. (13,14) This decline represents both the postponement and cancellation of planned care. For some time-sensitive procedures such as cleft lip and palate repair, delays could have a detrimental effect on long-term outcomes.

This study aimed to quantify the impact of the COVID-19 pandemic on the number and the timing of first primary cleft lip and/or palate (CLP) repair procedures using national longitudinal administrative hospital data from the English National Health Service (NHS). We hypothesised that there would be a reduction in the number of first primary CLP repair procedures during the COVID-19 pandemic year (2020/21) compared to the preceding year (2019/20). We defined the start of the first COVID-19 pandemic year as 1st April 2020 as this coincides closely with the official start of the first nationwide lockdown in England on 23rd March 2020. (15)

We also hypothesised that first primary CLP repair procedures would be delayed during the pandemic, so that the children at the time of surgery would be older than in the preceding year. Quantifying the extent of delays to surgery is important for planning of the future needs of these children.

METHODS

Study design

For peer review only - http://bmjopen.bmj.com/site/about/guidelines.xhtml

This is an observational study comparing the numbers of procedures, and the age at surgery for primary repair of cleft lip and/or palate at hospitals in England before (2019/20), or during (2020/21) the COVID-19 pandemic

Data source and study population

We used the Hospital Episode Statistics (HES), a national database including records of all episodes in NHS hospitals derived from administrative data.(16) HES records include diagnostic fields coded according to the International Classification of Diseases – 10th revision (ICD-10) (17) and procedure fields coded according to the Population Consensus and Surveys Classification of Interventions and Procedures – 4th revisions (OPCS-4).(18)

We identified all children born after 1st April 2014, who were considered to have an orofacial cleft because they had both a record with relevant diagnostic codes before their second birthday (or until 31st March, 2021, whatever came earlier) and a record with relevant CLP repair procedure codes before their fifth birthday (or until 31st March, 2021, whatever came earlier; see supplementary information 1 for code lists). We excluded children without a birth record in HES and children born from multiple pregnancies. Births recorded in HES represent 97% of all births in England. (19) Please see Supplementary Figure 1.

Outcome and patient characteristics

In the children identified with an orofacial cleft, we determined the date of their first primary CLP repair procedures with primary lip repair and primary palate repairs treated separately such that some children contributed more than one surgery. Secondary procedures were excluded from the analytical sample as other factors might influence their timing, including the timing of the primary surgery. We used diagnostic codes to distinguish four cleft types (see supplementary information 2 for code lists): cleft lip only (CL), cleft palate only (CP), unilateral cleft lip and palate (UCLP), bilateral cleft lip and palate (BCLP). We used

For peer review only - http://bmjopen.bmj.com/site/about/guidelines.xhtml

procedure codes to capture the type of surgery: primary lip repair (F031) and primary palate repair (F291). We also used ICD-10 codes to determine whether there were other additional congenital malformations (Supplementary information 3). (20,21) Quintiles of the national distribution of the 2019 Index of Multiple Deprivation (IMD) rankings of 32,844 Lower Super Output Areas (LSOA; areas with typically 1,500 inhabitants and 600 households) were used to categorise children into 5 groups according to their socioeconomic background. (22) Ethnicity was coded as White, and minority ethnicity including Black, Asian, mixed race, and other. Nine geographic regions of residence that correspond to the 9 regionally commissioned cleft services of England were derived from the LSOA.

Statistical analyses

We counted the number of first primary CLP repair procedures in 2020/21 and 2019/20 and calculated the relative difference between these numbers. Confidence intervals for these relative differences were calculated using the conditional method for testing differences between two Poisson means. (23) We used the Mantel-Haenszel test of homogeneity to investigate whether the difference between the number of procedures in 2019/20 and 2020/21 varied according to the children's characteristics.

To investigate changes in timing of first primary CLP repair procedures, we compared the mean age at the time of the first primary CLP procedures carried out in 2019/20 and 2020/21 with the t-test. Linear regression with interaction terms was used to test whether the difference between the means in 2019/20 and 2020/21 varied according to the children's characteristics.

Children with missing data on a specific characteristic were not included in the analyses involving that characteristic. A p-value <0.05 was considered to indicate a statistically significant result. All analyses were performed in Stata V.17 (Statacorp). (24)

For peer review only - http://bmjopen.bmj.com/site/about/guidelines.xhtml

Patient and public involvement

The ECHILD project undertakes regular patient and public involvement (PPI) including the acceptability of the use of de-identified data from healthcare and education settings, and research priorities for these datasets. Children and parents in our PPI workshops identified understanding the health and education impact of the pandemic on children with additional clinical needs (such as CLP) as a key priority for research.

RESULTS

Study population

We identified 6,438 children with a CLP procedure code recorded before the age of 5 between 1st April 2014 and 31st March 2021. Of these, 680 (10.6%) did not have a birth record or were born from multiple pregnancies and 257 (4.0%) did not have a CLP diagnostic code recorded before the age of 2. These children were therefore excluded (Supplementary Figure 1).

Number of first primary CLP repair procedures during the COVID-19 pandemic

In the remaining 5,501 children, we identified 774 first primary CLP procedures in 2020/21 corresponding to 321 first lip repair and 453 first palate repairs. This was in comparison to 942 procedures (408 lip repairs, 534 palate repairs) in 2019/20, a reduction of 17.8 % (95% confidence interval 9.5% to 25.4%; p <0.001; Table 1).

For peer review only

Table 1: Number of first primary cleft lip and palate repair procedures by year of surgery and the children's characteristics

	Number of procedures							
	Lip repair				Palate repairs			
**Year of surgery***	**2019/20**	**2020/21**	**Relative difference (95% CI)**	**p-value**	**2019/20**	**2020/21**	**Relative difference (95% CI)**	**p-value**
**All**	408	321	-21.32 (-32.24, -8.71)	0.0013	534	453	-15.17 (-25.32, -3.67)	0.0099
**Cleft type**	**(p=0.6389) ****				**(p=0.8277) ****			
Cleft lip only	157	116	-26.11 (-42.39, -5.47)	0.0131	—	—	—	—
Cleft palate only	—	—	—	—	284	233	-17.96 (-31.31, -2.09)	0.0249
Unilateral CLP	165	141	-14.54 (-32.24, 7.65)	0.1707	164	147	-10.37 (-28.75, 12.67)	0.3358
Bilateral CLP	86	64	-25.58 (-47.01, 4.05)	0.073	86	73	-15.12 (-38.73, 17.32)	0.3041
**Congenital malformations**	**(p=0.1867) ****				**(p=0.0210) ****			
No additional malformations	282	207	-26.60 (-38.95, -11.86)	0.0007	240	237	-1.25 (-17.82, 18.66)	0.8909
Additional malformations	126	114	-9.52 (-30.39, 17.50)	0.4396	294	216	-26.53 (-38.65, -12.12)	0.0005
**IMD quintile**	**(p=0.9045) ****				**(p=0.8962) ****			
Q1 (Most deprived)	94	86	-8.51 (-32.51, 23.90)	0.5522	127	114	-10.24 (-30.91, 16.51)	0.4034
Q2	79	61	-22.78 (-45.64, 9.22)	0.1293	117	98	-16.24 (-36.62, 10.49)	0.196
Q3	65	53	-18.46 (-44.36, 19.02)	0.2712	92	78	-15.22 (-38.11, 15.90)	0.2843
Q4	61	45	-26.23 (-50.95, 10.23)	0.1215	62	51	-17.74 (-44.35, 21.11)	0.3029
Q5 (least deprived)	47	36	-23.40 (-51.79, 20.79)	0.2299	55	57	3.51 (-42.28, 34.61)	0.8509
Missing	62	40	—	—	81	55	—	—
**Ethnicity**	**(p=0.7415) ****				**(p=0.3669) ****			
White/White British	327	253	-22.63 (-34.60, -8.55)	0.0021	414	341	-17.63 (-28.84, -4.71)	0.0079
Minority ethnicity	74	61	-17.57 (-42.25, 17.28)	0.2649	112	106	-5.36 (-28.12, 24.55)	0.6852
Missing	**7**	**7**	—	—	8	6	—	—
**Region**	**(p=0.4821) ****				**(p=0.3715) ****			
North-East	25	20	-8.70 (-51.94, 72.56)	0.766	20	30	34.37 (-17.37, 64.03)	0.1337

For peer review only - http://bmjopen.bmj.com/site/about/guidelines.xhtml

North-West	45	50	6.12 (-43.34, 38.61)	0.7596	75	60	-18.42 (-42.63, 15.61)	0.2349
Yorkshire	40	30	-21.05 (-52.76, 30.85)	0.3356	55	40	-28.30 (-54.01, 10.82)	0.1174
East Midlands	35	30	-14.29 (-49.17, 43.71)	0.5386	35	25	-27.78 (-58.13, 22.98)	0.2074
West Midlands	30	30	-3.12 (-42.83, 63.95)	0.9007	45	50	9.80 (-37.07, 40.79)	0.6137
East of England	30	25	-28.12 (-59.84, 26.73)	0.2288	40	30	-28.57 (-56.83, 16.87)	0.1597
London	50	35	-28.57 (-55.07, 12.49)	0.1284	60	60	0 (-45.48, 31.26)	1.0000
South-East	50	45	-9.61 (-40.40, 36.74)	0.6173	65	65	-1.54 (-31.38, 41.24)	0.9302
South-West	35	15	-57.14 (-78.25, -19.47)	0.0046	40	30	-21.95 (-52.42, 27.01)	0.2954
Missing	65	40	—	—	95	60	—	—
**Quarter**	**(p<0.0001) ****				**(p<0.0001) ****			
Q1 (Apr-Jun)	105	10	-90.48 (-95.56, -81.78)	<0.0001	136	27	-80.15 (-87.38, -69.83)	<0.0001
Q2 (Jul-Sep)	107	110	2.73 (-26.16, 28.10)	0.839	149	182	18.13 (-2.22, 34.52)	0.07
Q3 (Oct-Dec)	103	140	26.43 (4.43, 43.52)	0.0177	123	130	5.38 (-22.01, 26.66)	0.6606
Q4 (Jan-Mar)	93	61	-34.41 (-53.31, -8.43)	0.0099	126	114	-9.52 (-30.39, 17.50)	0.4396

* 2020/21: first year of COVID-19 pandemic; 2019/20: preceding year.

** – Mantel-Haenszel test for homogeneity, testing if the relative differences vary according to the children's characteristics.

Region figures rounded to the nearest 5 for disclosure control.

For peer review only

The reduction in the number of first lip repair observed in 2020/21 did not vary significantly according to the children's characteristics (p always >0.1 for cleft type, presence of additional anomalies, deprivation or ethnicity) or geographic region of residence. However, the reduction in lip repairs did vary according to quarterly period (p<0.0001).

The reduction in the number of the first primary palate repair procedures in 2020/21 varied according to quarterly period (p<0.0001) and was significantly larger for children with additional congenital malformations (p=0.0210).

No repair procedures were carried out in the first two months of the study period (1st April to 31st May 2020), primary cleft surgery resumed in the third month of the first quarter. The numbers of first primary procedures undertaken in the second and third quarters of 2020/21 (1st July and 31st December 2020) were higher and the number in the fourth quarter (between 1st January to 31st March) was lower than in the corresponding months in the preceding year. (Figure 1)

Timing of CLP surgeries before and during the COVID-19 pandemic

The mean age at the first primary lip repairs increased by 1.6 months (95% CI: 0.9, 2.2) in the first year of the COVID-19 pandemic compared to 2019/20 (see also Figure 2). This increase in age did not vary according to the children's characteristics (p always > 0.1). The largest increases in mean age of lip repairs were in the South-West 3.9 months (95% CI:), the East Midlands 3.5 months (95% CI:), and the first quarter of 2020/2021 3.4 months (95% CI:). (Table 2)

For peer review only - http://bmjopen.bmj.com/site/about/guidelines.xhtml

Table 2: Mean age at first primary CLP repair surgery by year of surgery and exposure variables

	Mean age at surgery in months (95% CI)							
	Lip repair				Palate repairs			
**Year of surgery**	**2019/20**	**2020/21**	**Difference**	**p-value**	**2019/20**	**2020/21**	**Difference**	**p-value**
**All**	5.72 (5.28, 6.15)	7.30 (6.84, 7.76)	1.58 (0.94, 2.22)	<0.0001	11.19 (10.60, 11.77)	11.81 (11.30, 12.33)	0.63 (-0.16, 1.42)	0.1209
**Cleft type**	**(p=0.4166) ****				**(p=0.9728) ****			
Cleft lip only	5.42 (4.77, 6.08)	7.14 (6.22, 8.05)	1.71 (0.62, 2.80)	0.0022	—	—	—	—
Cleft palate only	—	—	—	—	12.74 (11.92, 13.57)	13.50 (12.74, 14.25)	0.75 (-0.39, 1.88)	0.1961
Unilateral CLP	5.46 (4.83, 6.10)	7.34 (6.81, 7.87)	1.88 (1.04, 2.72)	<0.0001	8.96 (8.10, 9.82)	9.66 (9.08, 10.25)	0.70 (-0.35, 1.76)	0.1919
Bilateral CLP	6.75 (5.58, 7.93)	7.51 (6.31, 8.70)	0.75 (-0.94, 2.45)	0.3816	10.28 (8.73, 11.83)	10.77 (9.29, 12.24)	0.49 (-1.66, 2.64)	0.6525
**Congenital malformations**	**(p=0.8086) ****				**(p=0.4014) ****			
No additional malformations	5.02 (4.68, 5.37)	6.56 (6.14, 6.98)	1.53 (0.99, 2.07)	<0.0001	9.27 (8.64, 9.90)	10.47 (9.95, 10.98)	1.20 (0.38, 2.01)	0.0039
Additional malformations	7.27 (6.13, 8.42)	8.65 (7.61, 9.68)	1.37 (-0.17, 2.91)	0.0813	12.75 (11.86, 13.65)	13.29 (12.41, 14.18)	0.54 (-0.75, 1.83)	0.4102
**IMD quintile**	**(p=0.9259) ****				**(p=0.9099) ****			
Q1 (Most deprived)	5.42 (4.58, 6.26)	7.29 (6.35, 8.23)	1.87 (0.62, 3.12)	0.0035	10.38 (9.29, 11.48)	11.64 (10.40, 12.88)	1.26 (-0.38, 2.90)	0.132
Q2	6.34 (5.09, 7.59)	8.25 (6.68, 9.82)	1.91 (-0.05, 3.87)	0.0561	10.87 (9.83, 11.91)	11.47 (10.54, 12.40)	0.60 (-0.82, 2.01)	0.4067
Q3	5.85 (5.02, 6.69)	7.18 (6.55, 7.81)	1.33 (0.25, 2.40)	0.0164	10.42 (9.29, 11.55)	11.51 (10.52, 12.50)	1.09 (-0.42, 2.61)	0.1557
Q4	5.96 (4.52, 7.41)	7.06 (5.77, 8.35)	1.10 (-0.89, 3.09)	0.2772	11.22 (9.63, 12.80)	12.45 (10.62, 14.28)	1.23 (-1.15, 3.62)	0.3086
Q5 (least deprived)	4.91 (4.40, 5.41)	6.83 (5.95, 7.72)	1.93 (0.98, 2.87)	0.0001	11.78 (10.21, 13.35)	13.68 (12.05, 15.32)	1.91 (-0.34, 4.15)	0.0949
**Ethnicity**	**(p=0.9406) ****				**(p=0.9348) ****			
White/White British	5.54 (5.09, 6.00)	7.23 (6.72, 7.75)	1.69 (1.00, 2.38)	<0.0001	11.34 (10.64, 12.04)	11.94 (11.34, 12.54)	0.60 (-0.34, 1.54)	0.2129
Minority ethnicity	6.00 (5.06, 6.93)	7.63 (6.44, 8.82)	1.63 (0.15, 3.11)	0.0312	10.81 (9.80, 11.82)	11.49 (10.40, 12.57)	0.68 (-0.80, 2.15)	0.3659
**Region**	**(p=0.2113) ****				**(p=0.0022) ****			
North-East	4.92 (3.44, 6.41)	5.51 (4.30, 6.72)	0.59 (-1.29, 2.47)	0.5297	10.27 (8.15, 12.39)	9.76 (8.39, 11.13)	-0.51 (-2.86, 1.83)	0.6622
North-West	5.10 (4.08, 6.12)	7.13 (5.80, 8.46)	2.03 (0.36, 3.70)	0.0176	8.45 (7.36, 9.54)	11.02 (9.15, 12.88)	2.56 (0.51, 4.62)	0.0146
Yorkshire	4.87 (4.08, 5.66)	7.00 (5.55, 8.45)	2.13 (0.59, 3.66)	0.0072	9.68 (8.37, 10.99)	11.21 (8.32, 14.10)	1.53 (-1.31, 4.37)	0.2881

East Midlands	4.71 (3.95, 5.46)	8.16 (6.81, 9.51)	3.45 (2.00, 4.91)	<0.0001	9.15 (7.56, 10.74)	11.36 (9.30, 13.42)	2.21 (-0.29, 4.72)	0.0827
West Midlands	6.41 (4.02, 8.80)	8.33 (7.59, 9.06)	1.91 (-0.57, 4.40)	0.1283	10.34 (8.60, 12.08)	11.20 (10.43, 11.96)	0.86 (-0.95, 2.67)	0.3492
East of England	5.55 (4.47, 6.64)	5.88 (5.11, 6.65)	0.33 (-1.08, 1.73)	0.6439	12.12 (9.60, 14.64)	12.14 (10.05, 14.22)	0.01 (-3.40, 3.43)	0.9935
London	6.36 (4.50, 8.22)	6.88 (4.56, 9.21)	0.52 (-2.38, 3.43)	0.7203	12.04 (10.09, 13.99)	10.83 (10.02, 11.64)	-1.21 (-3.30, 0.87)	0.252
South-East	6.51 (5.02, 8.00)	8.34 (6.84, 9.84)	1.83 (-0.26, 3.92)	0.0858	14.16 (12.34, 15.97)	13.91 (12.61, 15.21)	-0.24 (-2.46, 1.97)	0.829
South-West	5.83 (5.18, 6.48)	9.72 (8.50, 10.93)	3.89 (2.66, 5.12)	<0.0001	10.96 (9.59, 12.33)	16.51 (14.42, 18.60)	5.55 (3.18, 7.92)	<0.0001
**Quarter**	**(p=0.6010) ****				**(p=0.5800) ****			
Q1 (Apr-Jun)	5.52 (4.53, 6.51)	8.92 (7.15, 10.69)	3.40 (0.14, 6.66)	0.0412	10.79 (9.85, 11.74)	12.17 (11.11, 13.24)	1.38 (-0.80, 3.56)	0.2142
Q2 (Jul-Sep)	6.19 (5.18, 7.21)	7.47 (6.87, 8.07)	1.27 (0.11, 2.44)	0.0322	12.33 (11.01, 13.64)	12.34 (11.58, 13.09)	0.01 (-1.44, 1.45)	0.9915
Q3 (Oct-Dec)	5.39 (4.66, 6.12)	7.02 (6.22, 7.82)	1.63 (0.51, 2.75)	0.0046	10.62 (9.46, 11.77)	11.80 (10.54, 13.07)	1.19 (-0.52, 2.90)	0.1721
Q4 (Jan-Mar)	5.76 (5.08, 6.43)	7.37 (6.16, 8.57)	1.61 (0.34, 2.88)	0.0133	10.82 (9.62, 12.03)	10.91 (10.11, 11.71)	0.08 (-1.38, 1.55)	0.9093

* 2020/21: first year of COVID-19 pandemic; 2019/20: preceding year.

** Test for interaction testing to see if the differences in mean age vary according to the children's characteristic

For peer review only

At national level, mean age at the first primary palate repair did not increase during 2020/21 (0.6 months, 95% CI: -0.2, 1.4) but there was some evidence of regional variation (p=0.0022) with the largest increases in mean age being observed in the South-West (5.6 months; 95% CI: 3.2 to 7.9) and North-West (2.6 months; 95% CI: 0.5, 4.6).

There was an increase in the proportion of lip repairs carried out after the age of 6 months from 19.4% (79/408; 95% CI: 15.6%, 23.5%) in 2019/20 to 57.9% (186/321; 95% CI: 52.3%, 63.4%) in 2020/21 ($p < 0.0001$). There was also a small but significant increase in the proportion of palate repairs carried out after the age of 12 months from 22.5% (120/534; 95% CI: 19.0%, 26.2%) in 2019/20 to 28.7% (130/453; 95% CI: 24.6%, 33.1%) in 2020/21 ($p = 0.025$).

DISCUSSION

This national study using routinely collected administrative hospital data of children born with an orofacial cleft in England found an 18% reduction in the number of first primary CLP repair procedures during the first year of the COVID-19 pandemic, as well as a delay of 1.6 months in the timing of the first primary lip repair procedure, compared to the preceding year. The largest difference was observed during the first quarter of the COVID-19 pandemic period. Also, the delay in the timing of procedures varied across the country with children residing in the South-West most affected.

The study has several strengths. First, the study population had excellent geographical coverage of England, reflecting all NHS hospitals. Secondly, by using both diagnosis and procedure codes to identify the study population, the impact of coding errors on the differences reported will have been reduced. Third, the relatively large study population made it possible to report differences in number and timing of first primary CLP repair surgeries undertaken by patient characteristics, by region and quarterly period.

For peer review only - http://bmjopen.bmj.com/site/about/guidelines.xhtml

Limitations include that for some children data items on their specific diagnosis were missing and when differences were compared by the children's characteristics, our results were based only on a complete-case analysis. It is unclear what impact this may have on the results reported as it is not known whether children with missing data were more or less likely to have delayed surgery for CLP repair than those with complete data. The use of ICD-10 and OPCS-4 codes may not capture more nuanced clinical information about individual diagnoses and procedures.

We showed that CLP repair surgery completely stopped in April and May 2020, which coincides with the start of the first national "lockdown" in England on 26th March 2020. This translated to a reduction in numbers of both primary lip repairs and primary palate repairs. Stakeholders will need to continue monitoring this as these reductions could have long term consequences (e.g. on speech development) and may have a time lag in their effect. The reduction in the number of first palate repair surgeries for children with additional congenital malformations was larger than for children without additional malformations, which may reflect deferred surgeries for children at higher risk of complications from COVID-19 (25). We also showed an increase in the age at first lip repair surgery, but no significant increase in the age at first palate repair. This may reflect clinical prioritisation of primary cleft palate repairs over cleft lip repairs. UK national guidance suggests that palate repair should be complete by 13 months of age (guidance palate repair at age 6 to 13 months) (UK guidance; 3-6 months of age for lip repairs)(26,27). However, we also showed that a significantly larger proportion of children had their first palate repair surgery after 12 months which might have long term consequences for education attainment as children who receive palate repairs after 13 months have been shown to have less favourable speech outcomes. (8,9)

We note that birth rates for England have slightly decreased over the study period, with a proportionate decline in the number of children born with CLP. (28) This may have had a

For peer review only - http://bmjopen.bmj.com/site/about/guidelines.xhtml

small impact on the number of expected operations but does not fully explain the observed reduction in number of procedures. The number of registrations recorded in CRANE for children born with cleft was approximately 7.7% lower in 2021 compared to 2020, (29) which is not sufficient to explain the relative reduction observed in our study (17.8% reduction). Furthermore, we observed delays in the timing of the lip and palate repairs (which is not dependent on the number of children born with a cleft), albeit the difference observed for palate repairs was not statistically significant.

Our study indicated that there were regional variations in the impact of the COVID-19 pandemic on the timing of first primary CLP repair procedures, which may reflect differences in the regions' influence on management decision making, resources, fragility, and capacity for recovery. The delay of almost 6 months seen in one region with other regions showing hardly any delay in the timing of first primary CLP repair procedures requires further investigation. COVID-19 pandemic associated delays in CLP repair have been reported in other countries, including a single-centre study in Peru where 172 patients demonstrated increases in age at the time of primary lip and palate repair. (30) Similarly, reduced volumes of procedures were recorded during the pandemic (relative to the pre-pandemic period) for Low and Middle Income Countries reporting to the Smile Train Express platform. (31)

This paper follows on from previous work which showed reductions in planned care during the pandemic and acts as a deeper dive into one specific type of planned care. (32) This work focuses on primary procedures which were given some prioritisation during the pandemic and as such might downplay its effect on wider cleft services. For example, secondary procedures such as Alveolar bone graft and secondary speech surgery are time sensitive but have less evidence supporting them. While the Federation of Specialist Surgical Associations Clinical Guide to Surgical Prioritisation during the COVID pandemic gave similar priority to primary and secondary cleft procedures, shop floor practicality may not necessarily have allowed

For peer review only - http://bmjopen.bmj.com/site/about/guidelines.xhtml

equal treatment. (33) Further work needs to be done to understand the full effect of the pandemic on all cleft surgery especially the more temporarily sensitive secondary cleft procedures (alveolar bone grafting and secondary speech surgery).

In conclusion, during the first year of the COVID-19 pandemic a larger proportion of children had their cleft repair surgery outside of the recommended timeframe (3 to 6 months for lip repair and 6 to 13 months for palate repair). Previous research has shown that late surgery may be associated with delays in speech development and the need for additional speech therapy.(8,9) Delayed surgery beyond 13 months is thought to affect articulation following cleft palate repair, and the resulting need for extra corrective speech therapy may contribute to additional absence from school, potentially affecting primary educational attainment.(7,34) Future research should therefore consider investigating the effect of delay in surgery on educational outcomes to model the long-term implications of the COVID pandemic.

FUNDING:

This project was funded by the National Institute for Health and Care Research (NIHR) Policy Research Programme. The views expressed are those of the author(s) and not necessarily those of the NIHR or the Department of Health and Social Care. This research was supported in part by the NIHR Great Ormond Street Hospital Biomedical Research Centre and the Health Data Research UK (grant no: LOND1), which is funded by the UK Medical Research Council and eight other funders. RB is supported by a UKRI Innovation Fellowship funded by the Medical Research Council (grant number MR/S003797/1).

AUTHOR CONTRIBUTIONS

For peer review only - http://bmjopen.bmj.com/site/about/guidelines.xhtml

JVDM and MHP developed the research question. DE, LMGL and RB operationalised the research question jointly with JVDM and MHP using data available via UCL Child Health Informatics team. DE undertook the analysis and drafted the manuscript. KF, SB, JM and CR provided essential clinical and contextual input into the study design and analysis, and interpretation of the results. All authors provided oversight and input to the final manuscript.

ACKNOWLEDGEMENTS

We thank Professor Ruth Gilbert and Professor Katie Harron for their support of the ECHILD project.

ETHICAL APPROVAL

Ethical approval was not required for this study because only de-identified routinely collected data were analysed.

CONFLICTS OF INTEREST

The authors have no conflicts of interest to declare

DATA SHARING STATEMENT

Data are available on request from NHS Digital and may not be shared by the authors.

REFERENCES

1. Cleft Lip and Palate Association (CLAPA), Cleft Registry and Audit Network (CRANE). CRANE 2020 Annual Report: Summary of findings for patients and parents/carers on children born with a cleft in England, Wales and Northern Ireland between January 2000 and December 2019. [Internet]. 2021 [cited 2022 Feb 3]. Available from: https://www.crane-database.org.uk/content/uploads/2021/03/CRANE-2020-AR_Patients-Parents-Carers-Summary_V1.pdf

For peer review only - http://bmjopen.bmj.com/site/about/guidelines.xhtml

2. Fitzsimons KJ, Copley LP, Van Der Meulen JH, Panagamuwa C, Deacon SA. Grommet Surgery in Children with Orofacial Clefts in England. Cleft Palate Craniofac J. 2017 Jan 1;54(1):80–9. https://doi.org/10.1597/15-047

3. Mildinhall S. Speech and Language in the Patient with Cleft Palate. Cleft Lip Palate. 2012;16:137–46. https://doi.org/10.1159/000337668

4. Stock N, Rumsey N. Living with a Cleft: Psychological Challenges, Support and Intervention. In: Cleft Lip and Palate: Diagnosis and Management. 2013. https://doi.org/10.1007/978-3-642-30770-6_45

5. Gallagher ER, Collett BR. Neurodevelopmental and Academic Outcomes in Children With Orofacial Clefts: A Systematic Review. Pediatrics. 2019 Jul 1;144(1):e20184027. https://doi.org/10.1542/peds.2018-4027

6. Fitzsimons KJ, Copley LP, Setakis E, Charman SC, Deacon SA, Dearden L, et al. Early academic achievement in children with isolated clefts: a population-based study in England. Arch Dis Child. 2018 Apr 1;103(4):356–62. https://doi.org/10.1136/archdischild-2017-313777

7. Fitzsimons KJ, Deacon SA, Copley LP, Park MH, Medina J, van der Meulen JH. School absence and achievement in children with isolated orofacial clefts. Arch Dis Child. 2021 Feb 1;106(2):154–9. https://doi.org/10.1136/archdischild-2020-319123

8. Shaffer AD, Ford MD, Losee JE, Goldstein J, Costello BJ, Grunwaldt LJ, et al. The Association Between Age at Palatoplasty and Speech and Language Outcomes in Children With Cleft Palate: An Observational Chart Review Study. Cleft Palate Craniofac J. 2020 Feb 1;57(2):148–60. https://doi.org/10.1177/1055665619882566

For peer review only

9. Lindeborg MM, Shakya P, Rai SM, Shaye DA. Optimizing speech outcomes for cleft palate. Curr Opin Otolaryngol Head Neck Surg. 2020 Aug;28(4):206–11. https://doi.org/10.1097/MOO.0000000000000635

10. Jiri B, Jana V, Michal J, Jiri K, Dana H, Miroslav T, et al. Successful early neonatal repair of cleft lip within first 8 days of life. Int J Pediatr Otorhinolaryngol. 2012 Nov 1;76(11):1616–26. https://doi.org/10.1016/j.ijporl.2012.07.031

11. Hammoudeh JA, Imahiyerobo TA, Liang F, Fahradyan A, Urbinelli L, Lau J, et al. Early Cleft Lip Repair Revisited: A Safe and Effective Approach Utilizing a Multidisciplinary Protocol. Plast Reconstr Surg – Glob Open. 2017 Jun;5(6):e1340. https://doi.org/10.1097/GOX.0000000000001340

12. Petráčková I, Zach J, Borský J, Černý M, Hacklová R, Tvrdek M, et al. Early and late operation of cleft lip and intelligence quotient and psychosocial development in 3–7years. Early Hum Dev. 2015 Feb 1;91(2):149–52. https://doi.org/10.1016/j.earlhumdev.2014.12.015

13. Isba R, Edge R, Jenner R, Broughton E, Francis N, Butler J. Where have all the children gone? Decreases in paediatric emergency department attendances at the start of the COVID-19 pandemic of 2020. Arch Dis Child. 2020 Jul 1;105(7):704–704. https://doi.org/10.1136/archdischild-2020-319385

14. Bottle A, Aylin P, Warner M, Propper C, Stoye G, Burn S. What happened to English NHS hospital activity during the COVID-19 pandemic? [Internet]. 2021 May [cited 2021 Nov 30]. Available from: https://ifs.org.uk/publications/15432

15. Timeline of UK government coronavirus lockdowns and restrictions [Internet]. The Institute for Government. 2021 [cited 2022 Sep 5]. Available from: https://www.instituteforgovernment.org.uk/charts/uk-government-coronavirus-lockdowns

For peer review only - http://bmjopen.bmj.com/site/about/guidelines.xhtml

16. Herbert A, Wijlaars L, Zylbersztejn A, Cromwell D, Hardelid P. Data Resource Profile: Hospital Episode Statistics Admitted Patient Care (HES APC). Int J Epidemiol. 2017 Aug 1;46(4):1093–1093i. https://doi.org/10.1093/ije/dyx015

17. WHO | ICD-10 online versions [Internet]. WHO. World Health Organization; [cited 2020 Apr 20]. Available from: http://www.who.int/classifications/icd/icdonlineversions/en/

18. OPCS classification of interventions and procedures. Version 4.8. Version 4.8 (2017). London: Stationery Office; 2017. Available from: https://digital.nhs.uk/data-and-information/information-standards/information-standards-and-data-collections-including-extractions/publications-and-notifications/standards-and-collections/dapb0084-opcs-classification-of-interventions-and-procedures

19. Harron K, Gilbert R, Cromwell D, Meulen J van der. Linking Data for Mothers and Babies in De-Identified Electronic Health Data. PLOS ONE. 2016 Oct 20;11(10):e0164667. https://doi.org/10.1371/journal.pone.0164667

20. Fitzsimons K, Hamilton M, van der Meulen J, Medina J, Wahedally M, Park M, et al. Range and Frequency of Congenital Malformations Among Children With Cleft Lip and/or Palate. Cleft Palate Craniofac J. 2022 Apr 5;10556656221089160. https://doi.org/10.1177/10556656221089160

21. Zylbersztejn A, Verfürden M, Hardelid P, Gilbert R, Wijlaars L. Phenotyping congenital anomalies in administrative hospital records. Paediatr Perinat Epidemiol. 2020 Jan;34(1):21–8. https://doi.org/10.1111/ppe.12627

22. Office of the Deputy Prime Minister, Neighbourhood Renewal Unit. The English indices of deprivation 2004. [Internet]. London: Office of the Deputy Prime Minister; 2004. Available from:

For peer review only

For peer review only - http://bmjopen.bmj.com/site/about/guidelines.xhtml

https://www.brent.gov.uk/media/323946/SD%2062b%20Indices%20of%20multiple%20deprivation%202004.pdf

23. Hilbe JM. Modeling Count Data [Internet]. Cambridge: Cambridge University Press; 2014 [cited 2022 Sep 5]. Available from: https://www.cambridge.org/core/books/modeling-count-data/BFEB3985905CA70523D9F98DA8E64D08

24. StataCorp. Stata Statistical Software. College Station, TX: StataCorp LLC; 2017.

25. COVID-19: guidance on protecting people defined on medical grounds as extremely vulnerable [Internet]. GOV.UK. [cited 2022 Dec 16]. Available from: https://www.gov.uk/government/publications/guidance-on-shielding-and-protecting-extremely-vulnerable-persons-from-covid-19

26. Federation of Surgical Specialty Association. Clinical Guide to Surgical Prioritisation During the Coronavirus Pandemic [Internet]. [cited 2022 Sep 5]. Available from: https://fssa.org.uk/_userfiles/pages/files/covid19/prioritisation_master_250920.pdf

27. Resumption of Cleft Services [Internet]. CLAPA. [cited 2022 Feb 3]. Available from: https://www.clapa.com/news-item/resumption-of-cleft-services/

28. Births in England and Wales: 2021, August 2022 [Internet]. Office for National Statistics. [cited 2023 Apr 17]. Available from: https://www.ons.gov.uk/peoplepopulationandcommunity/birthsdeathsandmarriages/livebirths/bulletins/birthsummarytablesenglandandwales/2021

29. Cleft Lip and Palate Association (CLAPA), Cleft Registry and Audit Network (CRANE). CRANE 2022 Annual Report: Summary of findings for patients and parents/carers on children born with a cleft in England, Wales and Northern Ireland between January 2000 and December 2021. [Internet]. 2022 [cited 2023 Apr 17]. Available from:

For peer review only

For peer review only - http://bmjopen.bmj.com/site/about/guidelines.xhtml

https://www.crane-database.org.uk/content/uploads/2022/12/CRANE-2022-Annual-Report_12Dec22.pdf

30. Rossell-Perry P, Gavino-Gutierrez A. Cleft Lip and Palate Surgery during COVID-19 Pandemic. Plast Reconstr Surg Glob Open. 2021 Jun 29;9(6):e3692. doi: 10.1097/GOX.0000000000003692

31. Vander Burg R, Agrawal K, Desai P, Desalu I, Donkor P. Impact of COVID-19 on elective cleft surgery in low- and middle-income countries. Plastic and Reconstructive Surgery – Global Open. 2021; 9(6): pe3656. DOI: 10.1097/GOX.0000000000003656

32. Etoori D, Harron KL, Grath-Lone LM, Verfürden ML, Gilbert R, Blackburn R. Reductions in hospital care among clinically vulnerable children aged 0–4 years during the COVID-19 pandemic. Arch Dis Child [Epub ahead of print]. 2022 Jun 20 [cited 2022 Sep 5]; https://doi.org/10.1136/archdischild-2021-323681

33. Clinical Guide to Surgical Prioritisation During the Coronavirus Pandemic [Internet]. FSSA. [cited 2022 Dec 15]. Available from: https://fssa.org.uk/covid-19_documents.aspx

34. Russell CJH, Fitzsimons KJ, Britton L, Van Eeden S, Butterworth S, Wahedally MHA, Park MH, Medina J, van der Meulen J, What Is The Optimal Timing Of Palate Repair For Speech Outcomes? Analysis Of Two Linked National Databases In England. [Oral presentation] CLEFT 2022 conference

For peer review only

Figure 1: Monthly numbers of first primary cleft lip repair and palate repair procedures in the first year of the COVID-19 period (between April 2020 and March 2021; red line) and the preceding year (between April 2019 and March 2020; blue line).

* Grey lines represent 5-year average (14/15 to 18/19) for historic comparison. Shaded areas represent lockdown periods (Lockdown 1: March 23 – June 23, 2020; Lockdown 2: November 5 – December 6, 2020; Lockdown 3: January 1 – March 8, 2021). CLP – Cleft lip and palate

For peer review only

Figure 2: Mean age at the first primary cleft lip and palate repair procedure in the first year of the COVID-19 pandemic (between April 2020 and March 2021; red square) and the preceding year (between April 2019 and March 2020; blue circle) with 95% confidence intervals.

Q1 – April – June; Q2 – July – September; Q3 – October – December; Q4 – January – March.

For peer review only

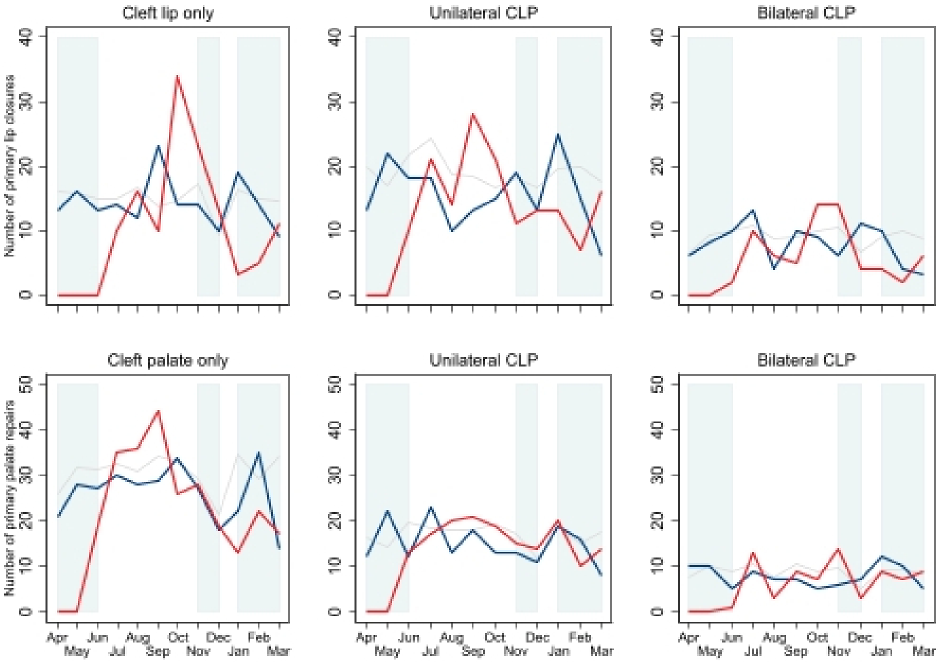

44x32mm (300 x 300 DPI)

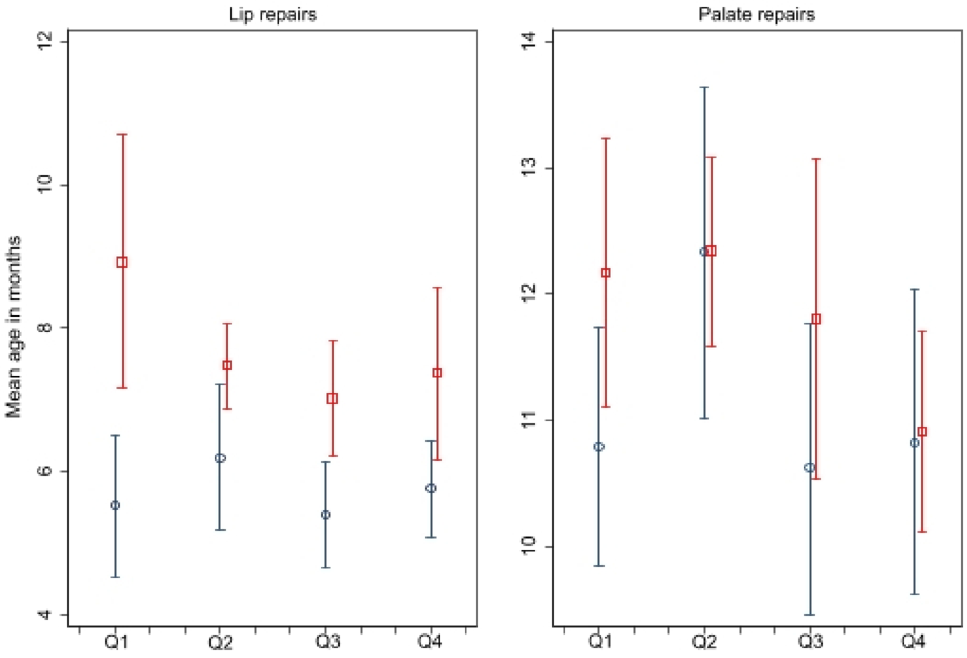

44x32mm (300 x 300 DPI)

SUPPLEMENTARY MATERIALS

Supplementary Figure 1: Flow chart showing inclusion criteria into the study

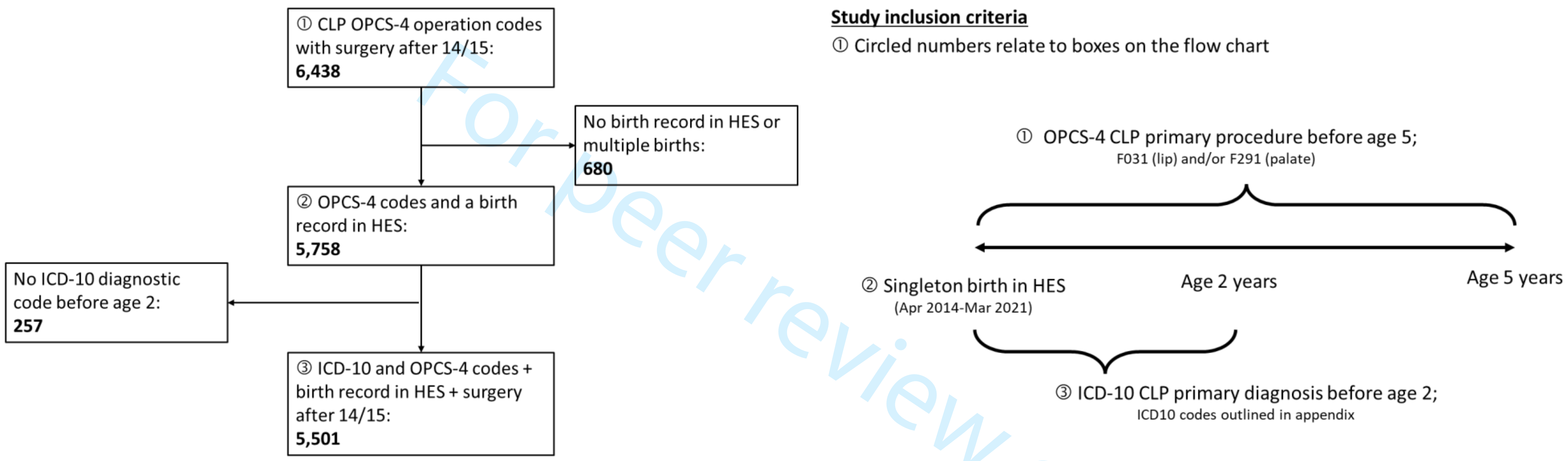

For peer review only - http://bmjopen.bmj.com/site/about/guidelines.xhtml

Supplementary information 1: OPCS-4 CLP surgery procedure code lists

OPCS-4 code	Surgery type
F031	Primary closure of cleft lip
F291	Primary palate repair

Supplementary information 2: Cleft lip and palate ICD-10 codes

ICD-10 codes	Cleft type
Q35x	Cleft lip
Q36x	Cleft palate
Q371, Q373, Q375, Q379	Unilateral cleft lip and palate
Q370, Q372, Q374, Q378	Bilateral cleft lip and palate

For peer review only - http://bmjopen.bmj.com/site/about/guidelines.xhtml

Supplementary information 3: Congenital anomalies code list

Code	Description
	Congenital malformations of the nervous system
Q00	Anencephaly and similar malformations
Q01	Encephalocele
Q02	Microcephaly
Q03	Congenital hydrocephalus
Q04	Other congenital malformations of brain
Q05	Spina bifida
Q06	Other congenital malformations of spinal cord
Q07	Other congenital malformations of nervous system
	Congenital malformations of eye, ear, face and neck
Q10	Congenital ptosis
Q11	Anophthalmos, microphthalmos and macrophthalmos
Q12	Congenital lens malformations
Q13	Congenital malformations of anterior segment of eye
Q14	Congenital malformations of posterior segment of eye
Q15	Other congenital malformations of eye
Q16	Congenital malformations of ear causing impairment of hearing
Q17	Other congenital malformations of ear

For peer review only

Q18	Other congenital malformations of face and neck
	Congenital malformations of the circulatory system
Q20	Congenital malformations of cardiac chambers and connections
Q21	Congenital malformations of cardiac septa
Q22	Congenital malformations of pulmonary and tricuspid valves
Q23	Congenital malformations of aortic and mitral valves
Q24	Other congenital malformations of heart
Q25	Congenital malformations of great arteries
Q26	Congenital malformations of great veins
Q27	Other congenital malformations of peripheral vascular system
Q28	Other congenital malformations of circulatory system
	Congenital malformations of the respiratory system
Q30	Congenital malformations of nose
Q31	Congenital malformations of larynx
Q32	Congenital malformations of trachea and bronchus
Q33	Congenital malformations of lung
Q34	Other congenital malformations of respiratory system
	Other congenital malformations of the digestive system
Q38	Other congenital malformations of tongue, mouth and pharynx
Q39	Congenital malformations of oesophagus
Q40	Other congenital malformations of upper alimentary tract
Q41	Congenital absence, atresia and stenosis of small intestine

For peer review only

For peer review only - http://bmjopen.bmj.com/site/about/guidelines.xhtml

Q42	Congenital absence, atresia and stenosis of large intestine
Q43	Other congenital malformations of intestine
Q44	Congenital malformations of gallbladder, bile ducts and liver
Q45	Other congenital malformations of digestive system
	Congenital malformations of the genital organs
Q50	Congenital malformations of ovaries, fallopian tubes and broad ligaments
Q51	Congenital malformations of uterus and cervix
Q52	Other congenital malformations of female genitalia
Q53	Undescended testicle
Q54	Hypospadias
Q55	Other congenital malformations of male genital organs
Q56	Indeterminate sex and pseudohermaphroditism
	Congenital malformations of the urinary system
Q60	Renal agenesis and other reduction defects of kidney
Q61	Cystic kidney disease
Q62	Congenital obstructive defects of renal pelvis and congenital malformations of ureter
Q63	Other congenital malformations of kidney
Q64	Other congenital malformations of urinary system
	Congenital malformations and deformations of the musculoskeletal system
Q65	Congenital deformities of hip
Q66	Congenital deformities of feet
Q67	Congenital musculoskeletal deformities of head, face, spine and chest

For peer review only - http://bmjopen.bmj.com/site/about/guidelines.xhtml

Q68	Other congenital musculoskeletal deformities
Q69	Polydactyly
Q70	Syndactyly
Q71	Reduction defects of upper limb
Q72	Reduction defects of lower limb
Q73	Reduction defects of unspecified limb
Q74	Other congenital malformations of limb(s)
Q75	Other congenital malformations of skull and face bones
Q76	Congenital malformations of spine and bony thorax
Q77	Osteochondrodysplasia with defects of growth of tubular bones and spine
Q78	Other osteochondrodysplasias
Q79	Congenital malformations of the musculoskeletal system, not elsewhere classified
	Other congenital malformations
Q80	Congenital ichthyosis
Q81	Epidermolysis bullosa
Q82	Other congenital malformations of skin
Q83	Congenital malformations of breast
Q84	Other congenital malformations of integument
Q85	Phakomatoses, not elsewhere classified
Q86	Congenital malformation syndromes due to known exogenous causes, not elsewhere classified
Q87	Other specified congenital malformation syndromes affecting multiple systems
Q89	Other congenital malformations, not elsewhere classified

For peer review only

For peer review only - http://bmjopen.bmj.com/site/about/guidelines.xhtml

	Chromosomal abnormalities, not elsewhere classified
Q90	Down syndrome
Q91	Edwards syndrome and Patau syndrome
Q92	Other trisomies and partial trisomies of the autosomes, not elsewhere classified
Q93	Monosomies and deletions from the autosomes, not elsewhere classified
Q95	Balanced rearrangements and structural markers, not elsewhere classified
Q96	Turner syndrome
Q97	Other sex chromosome abnormalities, female phenotype, not elsewhere classified
Q98	Other sex chromosome abnormalities, male phenotype, not elsewhere classified
Q99	Other chromosome abnormalities, not elsewhere classified

For peer review only

STROBE Statement—checklist of items that should be included in reports of observational studies

	Item No	Recommendation	Page No
**Title and abstract**	1	(*a*) Indicate the study's design with a commonly used term in the title or the abstract	1
		(*b*) Provide in the abstract an informative and balanced summary of what was done and what was found	2-3
**Introduction**			
Background/rationale	2	Explain the scientific background and rationale for the investigation being reported	3-4
Objectives	3	State specific objectives, including any prespecified hypotheses	4
**Methods**			
Study design	4	Present key elements of study design early in the paper	5
Setting	5	Describe the setting, locations, and relevant dates, including periods of recruitment, exposure, follow-up, and data collection	5-6
Participants	6	(*a*) *Cohort study*—Give the eligibility criteria, and the sources and methods of selection of participants. Describe methods of follow-up<br>*Case-control study*—Give the eligibility criteria, and the sources and methods of case ascertainment and control selection. Give the rationale for the choice of cases and controls<br>*Cross-sectional study*—Give the eligibility criteria, and the sources and methods of selection of participants	5-6
		(*b*) *Cohort study*—For matched studies, give matching criteria and number of exposed and unexposed<br>*Case-control study*—For matched studies, give matching criteria and the number of controls per case	
Variables	7	Clearly define all outcomes, exposures, predictors, potential confounders, and effect modifiers. Give diagnostic criteria, if applicable	5-6
Data sources/ measurement	8*	For each variable of interest, give sources of data and details of methods of assessment (measurement). Describe comparability of assessment methods if there is more than one group	4-5
Bias	9	Describe any efforts to address potential sources of bias	5
Study size	10	Explain how the study size was arrived at	5
Quantitative variables	11	Explain how quantitative variables were handled in the analyses. If applicable, describe which groupings were chosen and why	6
Statistical methods	12	(*a*) Describe all statistical methods, including those used to control for confounding	6
		(*b*) Describe any methods used to examine subgroups and interactions	6
		(*c*) Explain how missing data were addressed	7
		(*d*) *Cohort study*—If applicable, explain how loss to follow-up was addressed<br>*Case-control study*—If applicable, explain how matching of cases and controls was addressed<br>*Cross-sectional study*—If applicable, describe analytical methods taking account of sampling strategy	6
		(*e*) Describe any sensitivity analyses	

Continued on next page

For peer review only - http://bmjopen.bmj.com/site/about/guidelines.xhtml

**Results**			
Participants	13*	(a) Report numbers of individuals at each stage of study—eg numbers potentially eligible, examined for eligibility, confirmed eligible, included in the study, completing follow-up, and analysed	7 & Suppl Fig 1
		(b) Give reasons for non-participation at each stage	
		(c) Consider use of a flow diagram	
Descriptive data	14*	(a) Give characteristics of study participants (eg demographic, clinical, social) and information on exposures and potential confounders	Table 1
		(b) Indicate number of participants with missing data for each variable of interest	
		(c) *Cohort study*—Summarise follow-up time (eg, average and total amount)	
Outcome data	15*	*Cohort study*—Report numbers of outcome events or summary measures over time	
		Case-control study—Report numbers in each exposure category, or summary measures of exposure	
		Cross-sectional study—Report numbers of outcome events or summary measures	7, T1
Main results	16	(*a*) Give unadjusted estimates and, if applicable, confounder-adjusted estimates and their precision (eg, 95% confidence interval). Make clear which confounders were adjusted for and why they were included	7-10
		(*b*) Report category boundaries when continuous variables were categorized	7-10
		(*c*) If relevant, consider translating estimates of relative risk into absolute risk for a meaningful time period	
Other analyses	17	Report other analyses done—eg analyses of subgroups and interactions, and sensitivity analyses	7-10
**Discussion**			
Key results	18	Summarise key results with reference to study objectives	14
Limitations	19	Discuss limitations of the study, taking into account sources of potential bias or imprecision. Discuss both direction and magnitude of any potential bias	15
Interpretation	20	Give a cautious overall interpretation of results considering objectives, limitations, multiplicity of analyses, results from similar studies, and other relevant evidence	15-16
Generalisability	21	Discuss the generalisability (external validity) of the study results	14
**Other information**			
Funding	22	Give the source of funding and the role of the funders for the present study and, if applicable, for the original study on which the present article is based	17

*Give information separately for cases and controls in case-control studies and, if applicable, for exposed and unexposed groups in cohort and cross-sectional studies.

Note: An Explanation and Elaboration article discusses each checklist item and gives methodological background and published examples of transparent reporting. The STROBE checklist is best used in conjunction with this article (freely available on the Web sites of PLoS Medicine at http://www.plosmedicine.org/, Annals of Internal Medicine at http://www.annals.org/, and Epidemiology at http://www.epidem.com/). Information on the STROBE Initiative is available at www.strobe-statement.org.

For peer review only

For peer review only - http://bmjopen.bmj.com/site/about/guidelines.xhtml
